# Performance and explainability of feature selection-boosted tree-based classifiers for COVID-19 detection

**DOI:** 10.1016/j.heliyon.2023.e23219

**Published:** 2023-12-07

**Authors:** Jesús Rufino, Juan Marcos Ramírez, Jose Aguilar, Carlos Baquero, Jaya Champati, Davide Frey, Rosa Elvira Lillo, Antonio Fernández-Anta

**Affiliations:** a IMDEA Networks Institute, 28918, Madrid, Spain; b CEMISID, Universidad de Los Andes, Mérida, 5101, Venezuela; c CIDITIC, Universidad EAFIT, Medellín, Colombia; d Universidade do Minho and INESC TEC, Braga, Portugal; e Inria, Rennes, France; f Universidad Carlos III, Madrid, Spain

**Keywords:** COVID-19 detection, Explainability analysis, Gradient boosting classifiers, Random forest, Recursive feature elimination, Shapley values

## Abstract

In this paper, we evaluate the performance and analyze the explainability of machine learning models boosted by feature selection in predicting COVID-19-positive cases from self-reported information. In essence, this work describes a methodology to identify COVID-19 infections that considers the large amount of information collected by the University of Maryland Global COVID-19 Trends and Impact Survey (UMD-CTIS). More precisely, this methodology performs a feature selection stage based on the recursive feature elimination (RFE) method to reduce the number of input variables without compromising detection accuracy. A tree-based supervised machine learning model is then optimized with the selected features to detect COVID-19-active cases. In contrast to previous approaches that use a limited set of selected symptoms, the proposed approach builds the detection engine considering a broad range of features including self-reported symptoms, local community information, vaccination acceptance, and isolation measures, among others. To implement the methodology, three different supervised classifiers were used: random forests (RF), light gradient boosting (LGB), and extreme gradient boosting (XGB). Based on data collected from the UMD-CTIS, we evaluated the detection performance of the methodology for four countries (Brazil, Canada, Japan, and South Africa) and two periods (2020 and 2021). The proposed approach was assessed in terms of various quality metrics: F1-score, sensitivity, specificity, precision, receiver operating characteristic (ROC), and area under the ROC curve (AUC). This work also shows the normalized daily incidence curves obtained by the proposed approach for the four countries. Finally, we perform an explainability analysis using Shapley values and feature importance to determine the relevance of each feature and the corresponding contribution for each country and each country/year.

## 1 Introduction

During the COVID-19 pandemic, healthcare systems have faced significant challenges in developing surveillance strategies to monitor the spread of the disease. Specifically, these strategies require the collection of high-quality data almost in real-time [1]. In this regard, polymerase chain reaction (PCR) tests have been widely utilized to monitor the spread of the infectious disease. However, many factors affect the accuracy of PCR tests, including the timing of the test relative to the infection [2], the high rate of asymptomatic cases [3], and the limited availability of test kits [4]. To overcome these limitations, numerous approaches have been developed that make use of survey data to track pandemic indicators. For instance, [5] and [4] collected self-reported symptoms provided by individuals tested via PCR to evaluate the performance of COVID-19 detection methods. Similar approaches captured self-reported symptoms through smartphone apps to predict potential COVID-19 cases [6], [7], [8]. Social networks have also been used to publicize online questionnaires about symptoms, social behavior, and isolation measures [9].

Several methods have been developed for the detection of COVID-19-active cases based on individual features extracted from survey data. These methods can be categorized into three classes: prediction rules, logistic regression methods, and machine learning models [10]. Prediction rules identify active cases based on a specific set of symptoms. COVID-like illness (CLI) approaches approved by either the Centers for Disease Control and Prevention (CDC) or the World Health Organization (WHO) are the most representative prediction rules [11], [12], [13]. Additional prediction rules have been reported in [14], [2], [15]. Prediction rules were developed as a simple tool for making decisions about hospitalizations and managing healthcare resources efficiently (hospital beds and intensive care units) when antigen tests were not available. These rules typically consider a small number of symptoms with equal importance. On the other hand, methods based on logistic regression build a linear expression whose parameters represent the contribution of the reported features (symptom, gender, age group) [5], [6], [9], [16], [17], [18]. A reduced number of symptoms is also used in these techniques (usually less than five). Finally, machine learning models optimize supervised classifiers using multiple individual features to predict COVID-19 [1], [4]. Nevertheless, machine learning methods only take into account a limited number of features (symptoms, gender, and age) and ignore information provided by features such as vaccination acceptance, isolation measures, and local community information.

In April 2020, the University of Maryland (UMD), in collaboration with Facebook, launched the Global COVID-19 Trends and Impact Survey (UMD-CTIS), a large health surveillance system based on surveys [19], [1]. More precisely, the purpose of this study was to gather daily information from a representative sample of Facebook's Active User Base (FAUB), who were invited to participate in the survey. This instrument collected information about various COVID-19-related characteristics, including symptoms, PCR test outcomes, vaccination acceptance, isolation measures, local community information, mental health, and demographics. Questionnaires were translated into 56 languages, and data were collected from 114 countries/territories, reaching a wide range of social and economic groups. Note also that the UMD-CTIS data provide fine-grained coverage of pandemic trends, which permits estimating various health indicator trends for different regions.

### 1.1 Contributions

In this paper, we introduce a machine-learning methodology for detecting COVID-19 cases using tree-based supervised classifiers and feature selection strategies. In contrast to prediction rules and logistic regression models, the proposed methodology takes into account a wide range of individual characteristics for COVID-19 detection. For example, the proposed approach considers other factors besides symptoms, including demographics, vaccination acceptance, local community indicators, and isolation measures. As an alternative to previous machine learning approaches, the proposed approach utilizes a feature selection technique based on Shapley values to reduce dimensionality and minimize overfitting risk. Based on Shapley values, the optimal set of features is identified for the optimal balance between model complexity and detection accuracy [20]. Moreover, compared to prediction rules, supervised tree-based classifiers exhibit outstanding detection performance and allow us to recognize the relevant features contributing to detection tasks. We implemented six versions of the proposed methodology using three different classifiers: random forest (RF), light gradient boosting (LGB), and extreme gradient boosting (XGB). In addition, we evaluated the performance of the developed approach using UMD-CTIS datasets extracted from four countries: Brazil, Canada, Japan, and South Africa, for 2020 and 2021. The performance of the proposed methodology was compared to state-of-the-art techniques based on survey data. In general, our approach has outperformed other state-of-the-art methods in terms of different quality metrics including F1 score, sensitivity, specificity, and precision. Subsequently, we obtained the receiver operating characteristic (ROC) curves for the six versions of the proposed method and calculated the corresponding area under the ROC curves (AUC). As an illustrative example, we determined the normalized COVID-19 daily incidence using the proposed detection approach and compared the generated curves with those generated by official reports covering the four countries from April 2020 to June 2022. A further explanationability analysis has been performed in this study to identify the relevant input features and to outline how they contribute to the detection task [21], [22].

### 1.2 Related work

Recent studies have focused on the detection of COVID-19 using explainable machine-learning models. In particular, two machine learning techniques were discussed in [23], the multilayer perceptron artificial neural network and the decision tree. These techniques were used to predict the severity level of COVID-19 patients based on their medical history and laboratory test results. Moreover, a LIME approach was also used to evaluate the explainability of predictions produced by machine learning models. Furthermore, Girardi et al. [24] designed machine learning models (Random Forest, Neural Network, and Time Convolutional) to predict hospitalizations in COVID-19 positive-tested. Additionally, a study of the SHAP values to define the feature importance for the models in different scenarios reveals a high degree of variability across models.

In [25], an effective COVID-19 explanation was developed based on user-centered principles. More precisely, they discussed how to apply an interdisciplinary, user-centered approach based on Design Thinking to develop a prototype of a user-centered explanation regarding people's perception of COVID-19 vaccine development. In [26], machine learning and explainability methodologies were used to construct an aggravation risk score and analyze the effects of COVID-19 features. Age, chest CT severity, and biological variables such as CRP, oxygen saturation, and eosinophil counts were the most important factors. The work reported in [27] discussed the importance of self-organizing maps to interpret hospital data. Particularly, the COVID-19 epidemic was analyzed in detail to understand data patterns and topologies. They determine the most significant variables with networks and topological mapping, which solve this problem by mapping high-dimensional data into lower-dimensional representations based on the overall association.

For classification tasks of CT-COVID-19 images containing clinical findings of COVID-19 from 216 patients, Phongchit et al. [28] studied well-known neural network models (ResNet50V2, DenseNet169, Xception, and EfficientNet B4) to evaluate their performance and explainability. They concluded that the models producing the same COVID-19 classification result might rely on a large number of different features. This implication suggests that although we tend to select the model that performs best in metrics, in a clinical environment, it can be better to assess explanations from them. On the other hand, Aldhahi et al. [29] defined a method to train deep learning models in classifying COVID-19 chest X-rays from normal and pneumonia-related infections, using a training scheme that integrates the cyclic cosine annealing approach with cross-validation and uncertainty quantification. Additionally, they introduced an image processing technique to measure explainability based on ground truth. Ali et al. [30] develop a Convolutional neural network (CNN) model densely connected squeeze convolutional neural network for the classification of X-ray images of COVID-19, pneumonia, normal, and lung opacity patients. Then, to ensure model trust and explainability, they applied two explainable techniques, Grad-CAM and LIME. The goal of the work of Saxena et al. [31] was to detect disease in persons who had an X-ray image. Chest X-rays of COVID-19 patients, viral pneumonia patients, and healthy patients were obtained from different sources. These three groups were classified using deep learning and multiclass classification models. Then, they added a discussion about the explainability of the models. Li et al. [32] developed a multi-task learning framework in which COVID-19 diagnosis and multi-lesion recognition (segmentation of CT images) are achieved simultaneously. The framework is based on an explainable multi-instance multi-task network, which learns task-related features adaptively, and gives explicable diagnosis results by suggesting local CT images with lesions as additional evidence. Finally, severity assessment of COVID-19 and lesion quantification are carried out.

Other recent research in 2023 has continued to study the ability of machine learning models to analyze images of patients with COVID-19, but none consider aspects of explainability. For example, Kathamuthu et al. [33] used several enhanced CNN approaches using transfer learning to detect COVID-19 in chest computed tomography (CT) images. VGG16, VGG19, Densenet121, InceptionV3, Xception and Resnet50 are the basic models used in this work to apply transfer learning. Another work in the same line was proposed by Deeb et al. [34], who propose a CNN, called AdjCNet, that focuses on grayscale variations between adjacent areas within a CT image. The work of Ullah et al. [35] defines a multi-task semi-supervised learning (MTSSL) framework for performing COVID-19 detection in chest x-rays (CXR), which solves the problem of limited amount of labeled data in this domain. MTSSL uses auxiliary tasks for which adequate data are publicly available, specifically, pneumonia, lung opacity, and pleural effusion, which enrich the primary task of COVID-19 detection. MTSSL uses an unsupervised adversarial autoencoder (AAE) to learn and discriminate features and supervised classification networks for COVID-19 detection. Finally, Ershadi et al. [36] considered a special set of characteristics fusing clinical and image data to find treatment plans in groups of patients with COVID-19. They propose a hierarchical model based on expert knowledge to group patients, and then build classifier systems for each group. To design the proposed hierarchical model, they used a fuzzy C-mean (FCM) clustering for clustering tasks and an adaptive neuro-fuzzy inference system (ANFIS) classifier for classification tasks. As can be seen, recent works continue mainly along the same line of image processing, but without carrying out an explainability analysis of the results. Only in the work of Ershadi et al., an FCM-ANFIS approach is proposed that allows an explainability analysis process by using these white box techniques, but this is not considered in the article. To compare this work with the previous one, we proceeded to define four criteria, which are:

• **Criterion 1:** The work performs a feature selection stage.
• **Criterion 2:** The work carries out an explainability analysis using different approaches.
• **Criterion 3:** The work considers the feature explainability during the feature selection stage.
• **Criterion 4:** The work considers other factors besides symptoms for COVID-19 detection.

Table 1 shows the criteria covered by the different COVID-19 detection approaches. Notice that the first criterion is satisfied only by [23] and our methodology. In contrast, our approach is the only one that considers feature explainability as a primary consideration during the feature selection stage (third criterion). According to the second criterion, all of the studies meet this standard, which confirms that explainability is one of the most important aspects to consider when exploring medical machine-learning applications. The fourth criterion is met by several studies that utilize a variety of sources in addition to COVID-19 symptoms.

**Table 1:** Criteria covered by various COVID-19 detection approaches.

Method	Criterion			
	1	2	3	4
Gabbay et al. [23]	✓	✓	✗	✓
Girardi et al. [24]	✗	✓	✗	✗
Novak et al. [25]	✗	✓	✗	✗
Excoffier et al. [26]	✗	✓	✗	✓
Yu et al. [27]	✗	✓	✗	✗
Phongchit et al. [28]	✗	✓	✗	✗
Aldhahi et al. [29]	✗	✓	✗	✗
Ali et al. [30]	✗	✓	✗	✓
Saxena et al. [31]	✗	✓	✗	✓
Li et al. [32]	✗	✓	✗	✗
Ershadi et al. [36]	✗	✗	✗	✓
Our work	✓	✓	✓	✓

In general, X-ray images of the lungs have been studied for COVID-19 detection; but there are also works that consider other variables such as symptoms, among others. On the other hand, there is an effort to make these models explainable, particularly those based on X-ray images. However, there are no works that seek to select the features automatically (in our case, using recursively the Shapley values), and from there, develop detection models of COVID-19, nor an in-depth analysis of the behavior (both performance and explainability) of self-explaining methods (such as those based on decision trees). In our case, the analysis took into account both the total and the selected characteristics.

Unlike these approaches, our methodology makes COVID-19 predictions and analyzes the explainability of the trained models from survey data that includes a wide range of individual features. This collection strategy enables disease monitoring in almost real-time using limited healthcare resources. Additionally, our methodology selects features based on their explainability, which is determined by the Shapley values.

### 1.3 Paper organization

This paper is organized as follows. Section 2 describes the tree-based classifiers used to detect COVID-19-positive cases and the explainability analysis approaches used to identify relevant features. We introduce the methodology for detecting COVID-19-active cases in Section 3. Section 4 shows extensive results on the performance of the proposed methodology and the corresponding explainability analysis. The discussion generated by both the performance evaluation and the explainability analysis is summarized in Section 5.

## 2 Materials and methods

In this section, we describe the UMD-CTIS dataset, machine learning methods used to identify COVID-19-positive cases, and the feature selection technique used.

### 2.1 Machine learning methods

Machine-learning (ML) models used in this work are tree-based classifiers [37], [38]. Specifically, we will use random forest (RF), extreme gradient boosting (XGB), and light gradient boosting (LGB). These models provide rankings of the relevance of input variables, which serves as the basis for performing an explainability analysis process. Furthermore, tree-based models were selected due to their outstanding performance in several applications and the low training times when the input vector is high-dimensional [39], [40]. It should be noted that our feature selection stage involves iterative training steps that use a large number of variables, so this last aspect is of great relevance in our study. Now, each model is presented:

• **Random Forest (RF)**[41], [37], [38]: RF consists of a set of decision trees, each generated by a bagging algorithm. These trees form a “forest” of trees voting for a specific result. This algorithm uses bootstrapping to fit decision trees with sub-samples of the original dataset. For each tree, the algorithm uses averaging to improve predictive accuracy and control overfitting. In classification algorithms, the most common output will be chosen as the final output of the algorithm.
• **Extreme Gradient-Boosting (XGB)**[42], [37], [38]: It is a class of ensemble machine-learning algorithms that can be used for classification problems. Ensembles are constructed from decision-tree models. The trees are added one at a time to the ensemble, and fitted to correct for prediction errors made by previous models. This is done using data that could not be learned so far. This technique is known as boosting. Moreover, XGB applies a regularization technique to reduce overfitting.
• **Light Gradient-Boosting (LGB)**[42], [37], [38]: LGB is a gradient-boosting algorithm based on decision trees which decreases memory usage and improves model efficiency. It uses two techniques: the classic XGB based on Exclusive Feature Bundling, and the Gradient-driven One Side Sampling (GOSS). GOSS keeps those instances with large gradients (they will contribute more to information gain) and randomly drops those instances with small gradients to improve accuracy estimation. It is faster than the XGB algorithm because GOSS filters out the data instances to find a suitable split value. This makes the process faster.

### 2.2 Explainability analysis methods

In medicine, there is an increasing demand for AI approaches that are both efficient and transparent, as well as easily explainable by a human expert [21], [22]. Currently, it is difficult to find explanations as to why a result occurs or how a model describes the underlying biological process [43]. In COVID-19 studies that use machine learning models, explainable AI is urgently needed to understand and retrace the machine's decision-making process. It is critical, for example, to analyze the relationships between symptoms, age groups, gender, and COVID-19 cases. On the other hand, explaining, interpreting, or understanding, are synonymous in the context of explainable AI, and various approaches have been proposed [43]. As a first categorization, the explanator must describe the model or result (e.g., classification, prediction). Therefore, this classification is whether the explicability is *global* to provide insights into the inner workings of the entire model for some specific dataset or *local* for a single test input *x* and its corresponding result *y* [44], [43]. There are two types of explicability: *ante-hoc* consists of building it directly from the beginning of model creation (the model can be understood immediately), and *post-hoc* consists of building it after model creation using a technique to extract the explanation [44], [43].

Global explanators try to reveal certain properties of the model independently of results. An example is the tree-based approach (e.g., decision trees and ensembles of decision trees, such as RF) [41], [45], [42]. In this case, the information gained from a variable accumulated over all trees can be used as a relevance measure. Another example of a feature importance metric for tree-based methods is the feature's depth in the tree. Local explanations are only valid near a result. The classic methods relate the model result to the feature vector by ranking the explanatory power, i.e. the salience of each feature. There are two main families of methods. The first, *Attention Based Models*, examines the most promising parts of input features that lead to a certain output for a given task. For a given output, they try to find out whether input features with high attention weights were responsible for the outcome. The second is *feature-attribution approaches* that explicitly try to quantify the contribution of each individual feature to the results. In this work, we analyze a global ante-hoc approach based on the tree-based methods using the feature ranking provided by them and a local and post-hoc approach that extracts the feature importance for a given input based on the fact that output can be written as the sum of bias and single feature contributions (Shapley values).

• **SHapley Additive exPlanations (SHAP values)**[20], [46]**:** Shapley values are an example of local approaches [20], [46], which are created by means of a method from coalitional game theory, assuming that each feature value of the instance is a player in a game where the output is the payoff. Shapley values dispense the payoff among the features. The goal of SHAP is to explain the output of a model by computing the contribution of each feature to this output. For that, SHAP computes Shapley values. A Shapley value assigns each feature an importance value for a particular output to define the explanation. This value, for feature *i*, is the unified measure of additive feature attributions ($\phi_{i}$) [20], [46]:
  (1) $$\phi_{i}=\sum_{S\in F\setminus \left\{i\right\}}\frac{\left|S\right|!(M-\left|S\right|-1)!}{M!}[f_{S\cup \left\{i\right\}}(x_{S\cup \left\{i\right\}})-f_{S}(x_{S})]$$
  where *F* is the set of input features, *S* is a subset of input features, *M* is the number of input features and $f_{S}(.)$ represents the output function of the model (e.g. prediction). In this equation, $\sum_{S\in F\setminus \left\{i\right\}}\frac{\left|S\right|!(M-\left|S\right|-1)!}{M!}$ represents the weighted average of all possible subsets of *S* in *F*. In addition, this equation considers the difference between when this feature is present in the output ($f_{S\cup \left\{i\right\}}(x_{S\cup \left\{i\right\}}$) and when it is absent ($f_{S}(x_{S})$). With these SHAP values, we are able to select the variables that give the model the highest contribution. The calculation time increases exponentially according to the number of features. To avoid this, one solution is to determine contributions for only a few samples of the possible coalitions. We have used a sample of 100 to compute the importance of each feature using the kernel-explainer function.
• **Tree-based methods**[41], [45]**:** The explainability analysis for tree-based ML methods is possible by their capabilities to rank their features/variables. These methods make it possible to compute various feature/variable importance measures to be used in an explainability analysis. For example, MDA (mean decrease in accuracy) determines feature importance (ranking) as the mean decrease of accuracy over all predictions, when a given variable is permuted after training [41], [45]. Thus, MDA calculates the average decrease of accuracy against random permutations of feature values in different cases. The cases use a trained model, and each tree of the model is permuted along the *m*-th feature and the average of these differences for all decision trees gives the m-th feature's MDA. Also, it can use the Gini value, which measures the average gain of purity by splits of a given variable [41], [45]. If the variable is useful, then it tends to split labeled nodes into single classes. The permuted variables tend neither to increase nor to decrease node purities. Permuting a useful variable tends to yield a large decrease in mean Gini gain. Gini importance is normally inferior to variable importance because it is more unstable and biased.

Finally, in some cases, the large number of features could be a problem because of the noise produced by some of the variables or their low significance. There are some ML algorithms that already have some regularization mechanisms that reduce the number of features. However, the techniques being used in this study do not have any of these mechanisms. For this reason, we also use SHAP values as a feature-reduction technique that we apply to each of them. Ultimately, we end up with 6 different models, 3 of them with all the features (RF, LGBM and XGB), and 3 with the features selected according to the Shapley values (RF.SHAP, LGBM.SHAP and XGB.SHAP).

## 3 Experimental protocol

**Figure 1:**
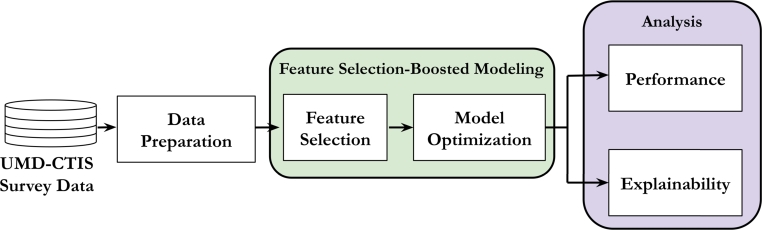
Flowchart illustrating the methodology for detecting COVID-19-active cases based on classifiers and feature selection and the corresponding performance evaluation and explainability analysis.

Fig. 1 illustrates the flowchart of the methodology for identifying COVID-19-positive cases from self-reported information using feature selection-boosted tree-based models. The datasets to be evaluated are extracted from the UMD-CTIS survey records. As shown in this figure, the methodology is divided into three stages: data preparation, feature selection-boosted modeling, and analysis. Data preparation involves data preprocessing and data understanding. Data preprocessing includes filtering techniques to extract the target datasets from UMD-CTIS data for the countries and periods of interest. Moreover, we perform a descriptive analysis of the dataset to gain more insight into the main characteristics of the study population. Feature selection and model optimization are the two steps in feature selection-boosted modeling. We implement a feature selection technique based on the RFE approach to reduce the number of variables without compromising performance. Then, the selected variables are used to optimize the tree-based supervised classification models. Finally, we conduct performance evaluation and explainability analysis from the outcomes yielded by the tree-based classifiers.

### 3.1 Dataset preparation

The University of Maryland (UMD), in partnership with Facebook, launched the Global COVID-19 Trends and Impacts Survey (UMD-CTIS), an extensive remote health surveillance system to monitor the COVID-19 pandemic evolution. More precisely, the UMD-CTIS collected self-reported data on approximately 120 indicators related to COVID-19, such as symptoms, age groups, gender, demographics, isolation measures, vaccination acceptance, and mental health, among others. In addition, the survey was run daily from April 23, 2020, to June 25, 2022 [1] (the questionnaire of the UMD-CTIS survey is shown in Supplementary Material 1). In this study, we extracted UMD-CTIS data from four countries: Brazil (BR), Canada (CA), Japan (JP), and South Africa (ZA). Geographic diversity and the availability of sufficient samples were considered when selecting these countries. Moreover, we considered data for two periods: 2020 (April 23 - December 31, 2020) and 2021 (January 1 - December 31, 2021). We selected these periods to observe the impact of vaccination campaigns on both the feature selection stage and the tree-based model optimization. Table 2 provides a summary of the population characteristics for the four countries and 2020 and 2021. As can be seen, there are 9,553,352 survey responses for all countries and periods (BR: 3,470,298, CA: 627,813, JP: 2,132,918, ZA: 329,528, for 2020; and BR: 1,669,105, CA: 282,914, JP: 918,147, ZA: 122,629, for 2021).

**Table 2:** Characteristics of the study population for the various countries and two non-overlapped periods (2020 and 2021).

Characteristic			Brazil		Canada		Japan		South Africa	
			2020	2021	2020	2021	2020	2021	2020	2021
1.	Number of responses		3470298	1669105	627813	282914	2132918	918147	329528	122629
2.	Tested symptomatic		83238	262683	8927	33997	4698	41010	7883	23038
	(a)	Training size	66590	210146	7142	27198	3758	32808	6306	18430
	(b)	Test size	16648	52537	1785	6799	940	8202	1577	4608
3.	Test outcome									
	(a)	Positive	44963	106471	838	3433	532	4011	2866	8459
	(b)	Negative	38275	156212	8089	30564	4166	36999	5017	14579
	(c)	TPR	54.02%	40.53%	9.39%	10.10%	11.32%	9.78%	36.35%	36.71%
	(d)	Minimum test size	1527	1482	523	558	617	542	1422	1428
4.	Gender									
	(a)	Female	45357	130235	5438	19472	1679	14283	3923	11291
	(b)	Male	24928	76689	2315	9824	2388	20791	2525	6730
5.	Age groups									
	(a)	18-24	8270	27474	1136	3248	179	871	739	1580
	(b)	25-34	19596	56227	2337	7172	577	3797	2252	4889
	(c)	35-44	21061	57452	1750	6688	997	7527	1801	4721
	(d)	45-54	13776	39122	1210	5215	1216	10413	1141	3878
	(e)	55-64	6968	22190	954	4478	828	8724	491	2124
	(f)	65-74	140	6016	308	2421	479	3529	1667	799
	(g)	75+	233	1025	126	825	66	846	27	230
6.	Average number of		4.33	3.80	3.38	3.07	2.92	2.49	3.82	3.95
	reported symptoms									
7.	Average number of		5.37	5.16	5.25	5.27	4.38	4.45	5.51	5.61
	reported symptoms									
	among positive									

Since survey data contain categorical data only, we first apply binary encoding to the dataset extracted from each country and period. Therefore, we have a column with binary elements for each potential response. For 2020 and 2021, binary encoding generated datasets with 417 and 614 columns, respectively. Surveys for 2021 include additional questions related to the vaccine campaigns. Then, we extracted samples from participants who had reported at least one symptom during the previous 24 hours and who had provided a test result within the past 14 days. Samples with symptomatic reports were selected to compare the performance of the proposed approach with respect to previously developed methods based on self-reported symptoms. In addition, we considered the samples with antigen test results to have a ground truth to train and test the detection models. For 2020, we analyzed 104,746 respondents (BR: 83,238, CA: 8,927, JP: 4,698, and ZA: 7,883) who reported at least one symptom in the last 24 hours and provided a test result within the past 14 days (Tested symptomatic). Similarly, we extracted data from 370,728 individuals in 2021 (BR: 262,683, CA: 33,997, JP: 41,010, and ZA: 23,038 ZA).

**Figure 2:**
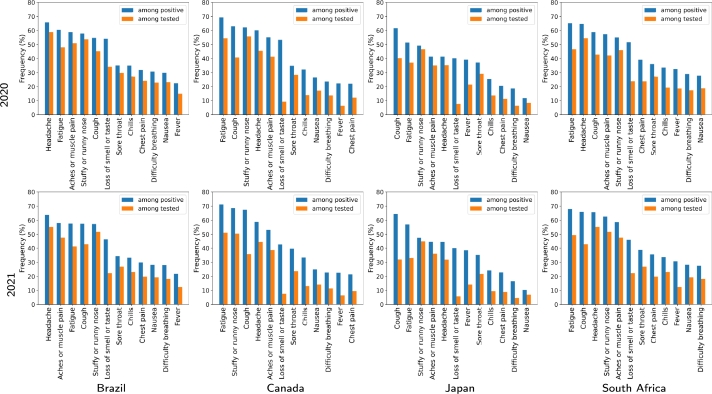
Rate of tested positives reporting a particular symptom in 2020 and 2021 for the four countries. Bar plots also show the percentage of tested symptomatic reporting each symptom.

Table 2 also includes the number of *positive* and *negative tests* among *tested symptomatic*, as well as the *test positive rate* (TPR), calculated as follows: TPR=(100×positive)/(Tested symptomatic), for each country and period. Notice that the TPR values obtained for Brazil and South Africa are at least three times larger than those yielded by Canada and Japan for 2020 and 2021. Table 2 displays information on other individual features such as gender, age group, the average number of reported symptoms per questionnaire, and the average number of reported symptoms per questionnaire among positives. In contrast to previous approaches that take into account a reduced set of individual features, our approach considers the full set of features collected by the UMD-CTIS questionnaires. Fig. 2 depicts the percentage of tested positives who reported a particular symptom for each country and period in descending order. In addition, Fig. 2 illustrates the corresponding rate of tested symptomatic reporting each symptom. As can be seen in this figure, fatigue is the most common symptom among positives, with the highest rates in the bar plots. The first conclusion is that, in general, the symptom patterns vary among countries and for 2020 and 2021. Finally, it is important to observe that the selected datasets are subject to potential bias sources, which may affect both the accuracy of the results as well as the identification of significant variables [1], [15]. As an example, the set of respondents is not a random sample of the population, since invitations to participate in the survey were sent to Facebook users. Furthermore, since CTIS-UMD provides COVID-19 signals based on self-reports, some indicators may differ from those obtained from more objective tests due to various sources of measurement error, such as recall bias and social desirability bias. However, we assume that these bias sources do not affect the results because of the amount of data used and they do not change rapidly over time, therefore, the signals reflect mean behavior during periods of interest (2020 and 2021).

### 3.2 Feature selection-boosted modeling

#### 3.2.1 Feature selection

As seen in Section 3.1, our methodology considers all variables extracted from each country and period. Notice that a large number of variables do not necessarily lead to performance improvement of the detection models and are typically associated with problems such as long training times and model overfitting [47]. Therefore, we include a feature selection stage to reduce the number of variables without compromising the detection performance. Moreover, this stage enables us to exclude irrelevant and redundant variables, thus simplifying models and boosting their explainability [48]. To implement the feature selection stage, we use the recursive feature elimination (RFE) approach proposed in [49] based on Shapley values. Algorithm 1 shows the pseudocode of the RFE method.

**Algorithm 1:**
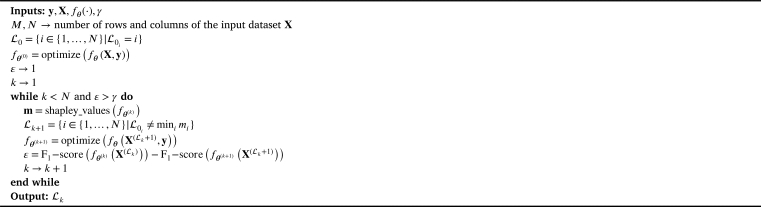
Recursive Feature Elimination (RFE) Algorithm.

As seen in Algorithm 1, the input dataset set **X** with dimensions M×N, where *M* is the number of samples and *N* is the number of features; the label vector **y** with *M* elements; the supervised machine learning model used for feature selection $f_{\theta }(\cdot )$; and the minimum performance loss *γ*. Initially, the procedure creates an index vector $L_{0}$ pointing to the selected features. Then, the machine learning model is optimized with the input dataset **X** and the label vector **y**. Moreover, the initial performance loss is set to one. For each iteration, the algorithm computes the Shapley values of the machine learning model and updates the set of indices by removing the least important feature. The machine learning model is then optimized based on the updated set of selected features. Notice that the model optimization stage is conducted using a hyperparameter optimization (HPO) based on the random search strategy. Moreover, due to that the classification models are based on trees, this optimization phase evaluates the performance of the trained models for different numbers of estimators (n_estimators) and numbers of leaves (num_leaves). The procedure computes the performance loss based on the F_1_-score. Finally, the algorithm returns the selected features after a loss criterion is achieved.

#### 3.2.2 Model optimization

We obtained the set of selected features for each country, period, and machine learning model. In this stage, we use the same supervised machine learning model as in the feature selection stage. For each country, period, and learning model, the extracted dataset was split into 100 partitions. For the training set, 80% of the samples were randomly selected from each partition. The remaining samples (20%) are included in the test set. The training and test sizes for the various countries and periods are shown in the third (2(a)) and fourth (2(b)) rows of Table 2. In addition, we estimate the minimum test sample size based on the TPR values for each country and period. We set the confidence interval at 95% and the marginal error at 2.50% [50], [51]. The ninth row (3(d)) of Table 2 displays the minimum test sample size for each country and period. As seen in this table, test sizes are larger than required for all countries and periods. For analyses with shorter durations (monthly, weekly, or daily), some countries and periods exhibit smaller test sample sizes than required. Therefore, for the selected countries, a yearly analysis is the minimum duration that satisfies the sample size requirements. Furthermore, as mentioned above, annual analyses (2020 and 2021) allow us to observe the impact of vaccination campaigns on the detection task and the identification of relevant variables. During the training phase, machine learning models applied an HPO based on the random search strategy. Then, we obtained the metrics results by evaluating the trained model on the test set. In general, performance results are calculated by averaging 100 realizations of the corresponding partitions, and the explainability analysis is performed on the model generating the best F_1_-score.

## 4 Results

### 4.1 Performance analysis

Table 3 illustrates different performance metrics in percentage and the 95% confidence intervals (CIs) generated by the various implementations of the proposed approach for Brazil, in 2020 and 2021. In particular, this table includes $F_{1}$-score, specificity, sensitivity, and precision obtained by the proposed techniques. A bold font and an underlined value indicate the best and second-best values for each metric and year. For Brazil 2020, the method based on the RF classification method generates the best performance results, i.e., $F_{1}$-score (RF: 84.24%, 95% CI: 84.19% to 84.30%), specificity (RF: 82.88%, 95% CI: 82.79% to 82.98%), sensitivity (RF: 83.43%, 95% CI: 83.35% to 83.51%), and precision (RF: 85.08%, 95% CI: 85.00% to 85.15%). In addition, the second best values are generated by the RF model optimized using the recursive feature elimination stage based on Shapley values: $F_{1}$-score (RF SHAP: 84.23%, 95% CI: 84.18% to 84.28%), specificity (RF SHAP: 82.87%, 95% CI: 82.78% to 82.97%), sensitivity (RF SHAP: 83.41%, 95% CI: 83.33% to 83.49%), and precision (RF SHAP: 85.07%, 95% CI: 84.99% to 85.15%). For 2021, equally, the RF and RF SHAP classifiers generate the best and second-best metric values, respectively. It is important to note that methods based on Shapley values, that apply a significant dimensionality reduction, exhibit a negligible performance loss in comparison with methods that do not include the recursive feature elimination step. Tables SM2, SM3, and SM4 in Supplemental material 2 show the performance metrics in percentage and the 95% CIs yielded by the proposed detection methods for Canada, Japan, and South Africa, respectively.

**Table 3:** Performance metrics in percentage and the 95% confidence intervals (CIs) obtained by the proposed COVID-19 detection methods for Brazil and for 2020 and 2021.

Year	Method	F_1_-scores	Specificity	Sensitivity	Precision
2020	RF	**84.24 (84.19 - 84.30)**	**82.88 (82.79 - 82.98)**	**83.43 (83.35 - 83.51)**	**85.08 (85.00 - 85.15)**
	XGB	80.56 (80.50 - 80.62)	78.96 (78.86 - 79.06)	79.59 (79.50 - 79.67)	81.57 (81.49 - 81.65)
	LGB	80.28 (80.22 - 80.34)	78.53 (78.43 - 78.63)	79.37 (79.29 - 79.44)	81.22 (81.14 - 81.30)
	RF SHAP	84.23 (84.18 - 84.28)	82.87 (82.78 - 82.97)	83.41 (83.33 - 83.49)	85.07 (84.99 - 85.15)
	XGB SHAP	80.57 (80.51 - 80.63)	78.97 (78.87 - 79.07)	79.59 (79.51 - 79.66)	81.58 (81.50 - 81.66)
	LGB SHAP	80.26 (80.21 - 80.32)	78.53 (78.42 - 78.64)	79.34 (79.26 - 79.41)	81.21 (81.13 - 81.30)

2021	RF	**80.43 (80.39 - 80.47)**	**91.40 (91.37 - 91.43)**	**75.74 (75.68 - 75.80)**	**85.74 (85.68 - 85.79)**
	XGB	73.89 (73.85 - 73.94)	88.75 (88.71 - 88.79)	68.25 (68.19 - 68.31)	80.55 (80.48 - 80.61)
	LGB	73.50 (73.45 - 73.54)	88.54 (88.50 - 88.58)	67.86 (67.80 - 67.91)	80.16 (80,09 - 80.23)
	RF SHAP	79.11 (79.07 - 79.15)	90.15 (90.11 - 90.18)	74.90 (74.84 - 74.96)	83.85 (83.79 - 83.91)
	XGB SHAP	72.53 (72.49 - 72.58)	88.26 (88.22 - 88.30)	66.69 (66.62 - 66.76)	79.50 (79.43 - 79.57)
	LGB SHAP	73.49 (73.44 - 73.53)	88.54 (88.50 - 88.58)	67.84 (67.78 - 67.90)	80.16 (80.10 - 80.22)

**Figure 3:**
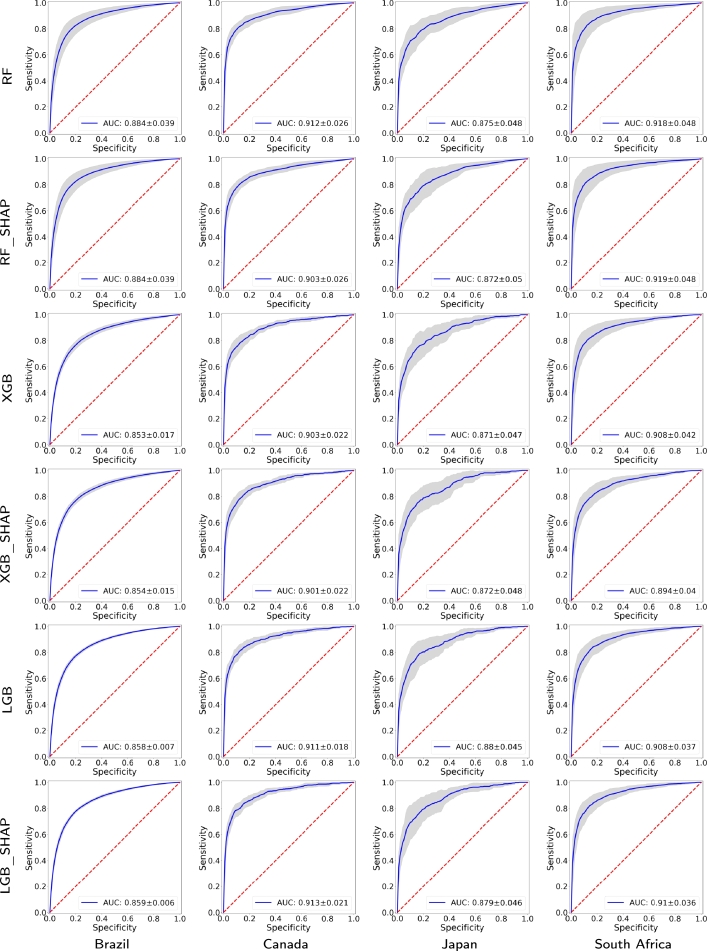
ROC curves and their 95% confidence intervals for the four countries and for 2020 using the proposed approach with different classifiers. The AUC value is included in each ROC curve.

Fig. 3 presents the receiver operating characteristic (ROC) curves and 95% CIs produced by the implemented machine learning models for the four countries and 2020. More precisely, each ROC curve is derived by averaging ten realizations of the respective experiment, where different training and test sets are randomly generated at each trial. The training set contains 80% of the samples, while the test set contains the 20% remaining ones. Every ROC curve includes the area under the ROC curve (AUC) and its 95% CI. For 2020, the detection methods obtaining the best auROCs for each country are Brazil (RF: 0.884, 95% CI: 0.845−0.923), Canada (LGB_Shap: 0.913, 95% CI: 0.892−0.934), Japan (LGB: 0.880, 95% CI: 0.835−0.925), and South Africa (RF_Shap: 0.919, 95% CI: 0.871−0.967). Note that the lowest AUC value is obtained for Brazil (XGB_Shap: 0.854, 95% CI: 0.839−0.869).

**Figure 4:**
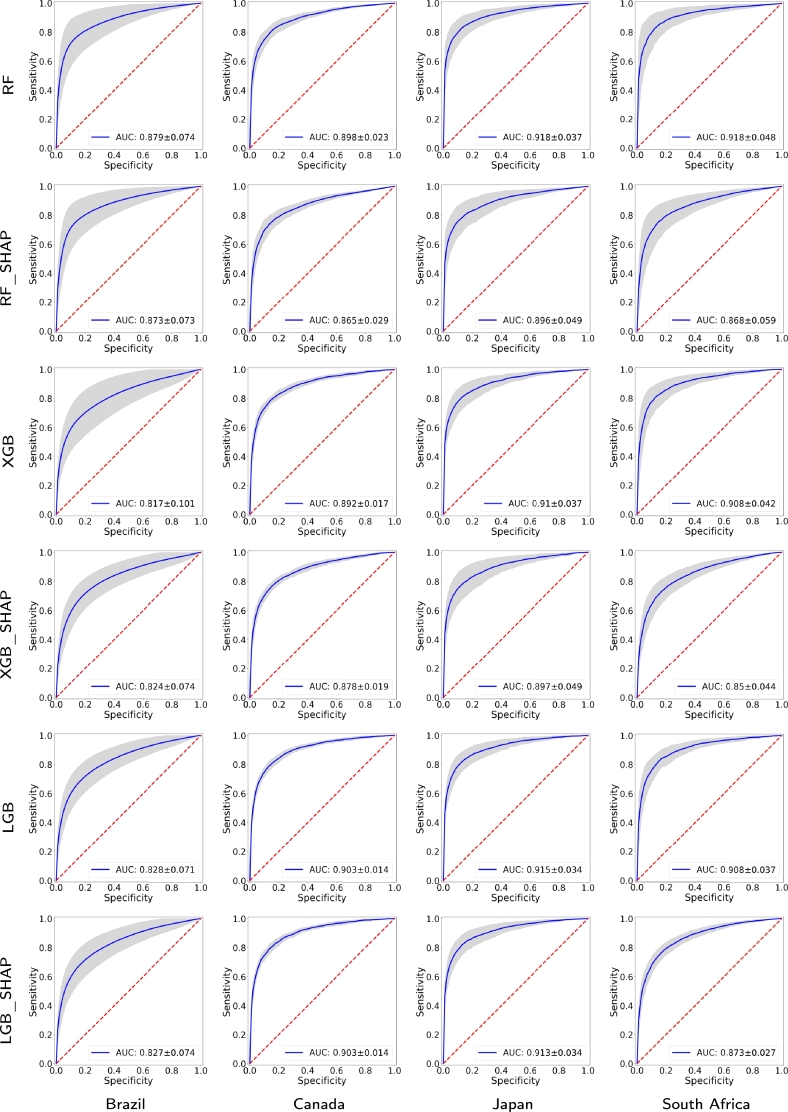
ROC curves and their 95% confidence intervals for the four countries and for 2021 using the proposed approach with different classifiers. The AUC value is included in each ROC curve.

On the other hand, Fig. 4 illustrates the ROC curves and their 95% CIs obtained by the proposed COVID-19 detection models for the four countries and 2021. The detection methods yielding the best AUC values for every country are Brazil (RF: 0.879, 95% CI: 0.805−0.953), Canada (LGB: 0.903, 95% CI: 0.889−0.917), Japan (RF: 0.918, 95% CI: 0.881−0.955), and South Africa (RF: 0.918, 95% CI: 0.870−0.966). For 2021, the lowest AUC value is also obtained for Brazil (XGB: 0.817, 95% CI: 0.716−0.918).

**Figure 5:**
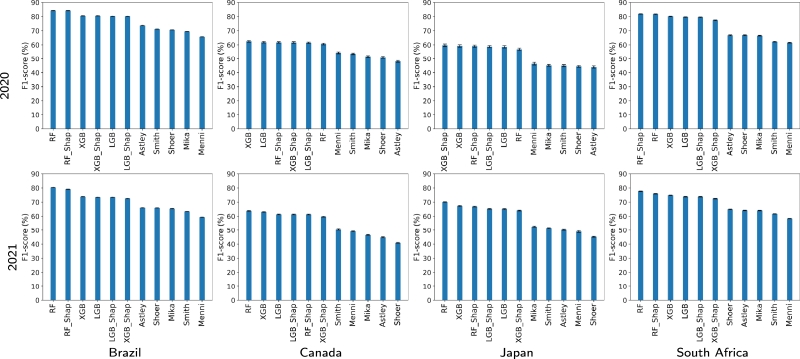
F_1_-scores and the 95% CIs yielded by various COVID-19 detection methods for the four countries and for 2020 and 2021.

Fig. 5 displays the $F_{1}$-scores and the corresponding 95% CIs obtained by different COVID-19 detection methods for the four countries and for 2020 and 2021. Notice that $F_{1}$-scores are presented in descending order to identify the best performance. Particularly, we display the $F_{1}$-scores produced by the detection methods based on RF, XGB, LGB, RF_Shap, XGB_Shap, and LGB_Shap. For comparison purposes, we also included the $F_{1}$-scores obtained by previously reported detection techniques such as Menni [6], Smith [5], Shoer [16], Mika [17], and Astley [1]. In Supplemental Material 2, Table SM1 shows the numerical values of the $F_{1}$-scores and the 95% CIs obtained by the different detection methods for the four countries and for 2020 and 2021. Specifically, for 2020, the detection methods that yield the best $F_{1}$-scores for every country are: Brazil (RF: 84.24%, 95% CI: 84.19% to 84.29%), Canada (XGB: 62.53%, 95% CI: 61.98% to 63.09%), Japan (XGB_Shap: 59.70%, 95% CI: 58.82% to 60.57%), and South Africa (RF_Shap: 81.88%, 95% CI: 81.68% to 82.09%). For 2021, the methods yielding the best $F_{1}$-scores for every country are: Brazil (RF: 80.43%, 95% CI: 80.39% to 80.47%), Canada (RF: 63.80%, 95% CI: 63.52% to 64.08%), Japan (RF: 70.11%, 95% CI: 69.84% to 70.37%), and South Africa (RF: 77.69%, 95% CI: 77.53% to 77.85%). It is worth noting that the proposed COVID-19 detection methods outperform previously reported techniques for the four countries and for the two periods under test. $F_{1}$ scores for 2020 and 2021 are presented in Figure SM1 in the Supplemental Material to compare the performance of each detection method across the countries under test. As shown in this figure, each method generates the best $F_{1}$ scores for Brazil or South Africa. Table 2 highlights that these countries exhibit TPR values that are at least three times larger than those of Canada and Japan.

The ANOVA test was conducted to assess whether there are statistically significant differences in performance among all detection methods based on the work [52]. Specifically, the ANOVA test was applied to the results of 100 realizations of the test set. Statistically significant differences were observed between the performance of the proposed COVID-19 detectors for 2020 and 2021 for all countries. As an illustrative example, Table 4 displays the results computed by the ANOVA test for five classification metrics ($F_{1}$-score, sensitivity, specificity, precision, and AUC) for the four countries and for 2020 and 2021. Furthermore, we used the Friedman test to compare the performance yielded by the proposed classifiers [53]. The Friedman test was also applied to the results of the 100 realizations of the test set. It is worth noting that this test shows statistically significant performance differences between the proposed machine learning models, with p<0.001 for all countries for all metrics, for 2020 and 2021. Additionally, a pairwise analysis was conducted using the Wilcoxon signed-rank test and different significance levels: α=0.05, α=0.01, and α=0.001 [52]. The pairwise comparison results using different significance levels for the AUC for the four countries in 2020 are displayed in Table 5. The Wilcoxon signed-rank test shows statistically significant differences in the AUC performance between RF and LGB, RF and LGB_SHAP, RF and XGB, and RF and XGB_SHAP. On the other hand, this test does not find statistically significant differences between RF and RF_SHAP, LGB and LGB_SHAP, and XGB and XGB_SHAP. Thus, notice that RF_SHAP, LGB_SHAP, and XGB_SHAP models, which are built using a reduced set of variables generated by the feature selection stage, exhibit similar performances to those yielded by RF, LGB, and XGB classifiers.

**Table 4:** Results of the ANOVA test for five classification metrics for the four countries for 2020 and 2021.

	Classification	Brazil		Canada		Japan		South Africa	
Year	Metric	F	p	F	p	F	p	F	p
2020	F_1_-score	4874.00	*p* < 0.001	5.01	*p* < 0.001	5.31	*p* < 0.001	234.10	*p* < 0.001
	Sensitivity	2789.36	*p* < 0.001	38.80	*p* < 0.001	28.89	*p* < 0.001	116.71	*p* < 0.001
	Specificity	1817.85	*p* < 0.001	114.33	*p* < 0.001	76.30	*p* < 0.001	79.14	*p* < 0.001
	Precision	2202.13	*p* < 0.001	75.64	*p* < 0.001	38.29	*p* < 0.001	110.62	*p* < 0.001
	AUC	4894.43	*p* < 0.001	27.88	*p* < 0.001	19.61	*p* < 0.001	222.14	*p* < 0.001

2021	F_1_-score	24479.56	*p* < 0.001	103.08	*p* < 0.001	245.99	*p* < 0.001	429.85	*p* < 0.001
	Sensitivity	17558.30	*p* < 0.001	73.83	*p* < 0.001	128.96	*p* < 0.001	255.35	*p* < 0.001
	Specificity	3849.87	*p* < 0.001	221.76	*p* < 0.001	166.94	*p* < 0.001	184.18	*p* < 0.001
	Precision	6395.48	*p* < 0.001	204.08	*p* < 0.001	210.32	*p* < 0.001	230.96	*p* < 0.001
	AUC	26173.41	*p* < 0.001	76.38	*p* < 0.001	156.77	*p* < 0.001	460.19	*p* < 0.001

**Table 5:** Results of the Wilcoxon signed-rank test using different significance levels for the AUC metric for the four countries in 2020.

	Brazil				Canada				Japan				South Africa			
Method Pairs	*p*	p<0.05	p<0.01	p<0.001	*p*	p<0.05	p<0.01	p<0.001	*p*	p<0.05	p<0.01	p<0.001	*p*	p<0.05	p<0.01	p<0.001
RF vs RF_SHAP	0.2342	✗	✗	✗	0.0000	✓	✓	✓	0.0000	✓	✓	✓	0.0119	✓	✗	✗
RF vs LGB	0.0000	✓	✓	✓	0.0000	✓	✓	✓	0.0000	✓	✓	✓	0.0000	✓	✓	✓
RF vs LGB_SHAP	0.0000	✓	✓	✓	0.0000	✓	✓	✓	0.0000	✓	✓	✓	0.0000	✓	✓	✓
RF vs XGB	0.0000	✓	✓	✓	0.0000	✓	✓	✓	0.0000	✓	✓	✓	0.0000	✓	✓	✓
RF vs XGB_SHAP	0.0000	✓	✓	✓	0.0000	✓	✓	✓	0.0000	✓	✓	✓	0.0000	✓	✓	✓
RF_SHAP vs LGB	0.0000	✓	✓	✓	0.0008	✓	✓	✓	0.0168	✓	✗	✗	0.0000	✓	✓	✓
RF_SHAP vs LGB_SHAP	0.0000	✓	✓	✓	0.0069	✓	✓	✗	0.1061	✗	✗	✗	0.0000	✓	✓	✓
RF_SHAP vs XGB	0.0000	✓	✓	✓	0.0000	✓	✓	✓	0.2070	✗	✗	✗	0.0000	✓	✓	✓
RF_SHAP vs XGB_SHAP	0.0000	✓	✓	✓	0.0000	✓	✓	✓	0.0082	✓	✓	✗	0.0000	✓	✓	✓
LGB vs LGB_SHAP	0.3154	✗	✗	✗	0.8615	✗	✗	✗	0.3358	✗	✗	✗	0.0837	✗	✗	✗
LGB vs XGB	0.0000	✓	✓	✓	0.0000	✓	✓	✓	0.0000	✓	✓	✓	0.0000	✓	✓	✓
LGB vs XGB_SHAP	0.0000	✓	✓	✓	0.0006	✓	✓	✓	0.0000	✓	✓	✓	0.0000	✓	✓	✓
LGB_SHAP vs XGB	0.0000	✓	✓	✓	0.0000	✓	✓	✓	0.0005	✓	✓	✓	0.0000	✓	✓	✓
LGB_SHAP vs XGB_SHAP	0.0000	✓	✓	✓	0.0007	✓	✓	✓	0.0000	✓	✓	✓	0.0000	✓	✓	✓
XGB vs XGB_SHAP	0.6180	✗	✗	✗	0.0388	✓	✗	✗	0.0327	✓	✗	✗	0.0000	✓	✓	✓

**Figure 6:**
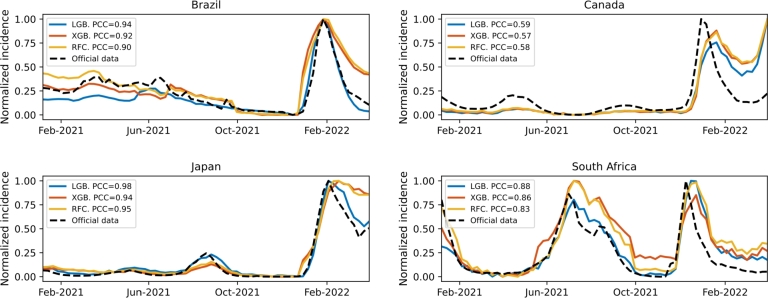
Normalized daily incidence curves generated by the proposed detection methods based on RF, LGB, and XGB. The normalized daily incidence curve determined from official reports is also displayed for each country.

Finally, to evaluate the proposed COVID-19 detection methods in a practical problem, we estimate the normalized COVID-19 daily case curves for the four countries from January 1, 2021, to June 25, 2022. Using the proposed detection methods based on RF, LGB, and XGB, we first identify the daily COVID-19 active cases in the interval of interest. Afterward, the estimated daily case curves are normalized with respect to the maximum value. A comparison of the daily case curves obtained by the proposed detection methods for the four countries is shown in Fig. 6. In addition, we include the normalized COVID-19 daily case curve provided by the respective national healthcare system for comparison. In each country, we present a Pearson correlation coefficient between the curve obtained by each proposed detection methodology and the curve provided by the national healthcare system. The best correlation coefficients are generated by the proposed approach based on the LGB classifier, i.e., Brazil (LGB: 0.94), Canada (LGB: 0.59), Japan (LGB: 0.98), and South Africa (LGB: 0.88). In general, the estimated curves follow the trends reported in official statistics for Brazil, Canada, Japan, and South Africa. For the winter of 2022, all the curves present some difficulty in following the trends of the official ones. Something important to highlight to explain this is that the UMD-CTIS collected information on the same variables across the entire period between April 2020 and June 2022, with a few minor modifications. However, these variables did not capture information on different indicators related to the dynamic behavior of the pandemic, such as the loss of effectiveness of vaccines over time or the emergence of new variants such as Omicron [54], which affected, in turn, the accuracy of the corresponding estimates. As an important note, the daily case curves generated by these detection methods have been used by the CoronaSurveys Project (https://coronasurveys.org), a collaboration between several academic institutions aimed at providing global pandemic surveillance based on surveys, to estimate daily active cases for more than 150 countries/territories [55].

### 4.2 Explainability analysis

This work considers a local and post-hoc approach based on Shapley values and a global ante-hoc approach based on the RF because of its superior accuracy with respect to the other tree-based methods considered in this work.

#### 4.2.1 Explainability analysis using Shapley values

**Figure 7:**
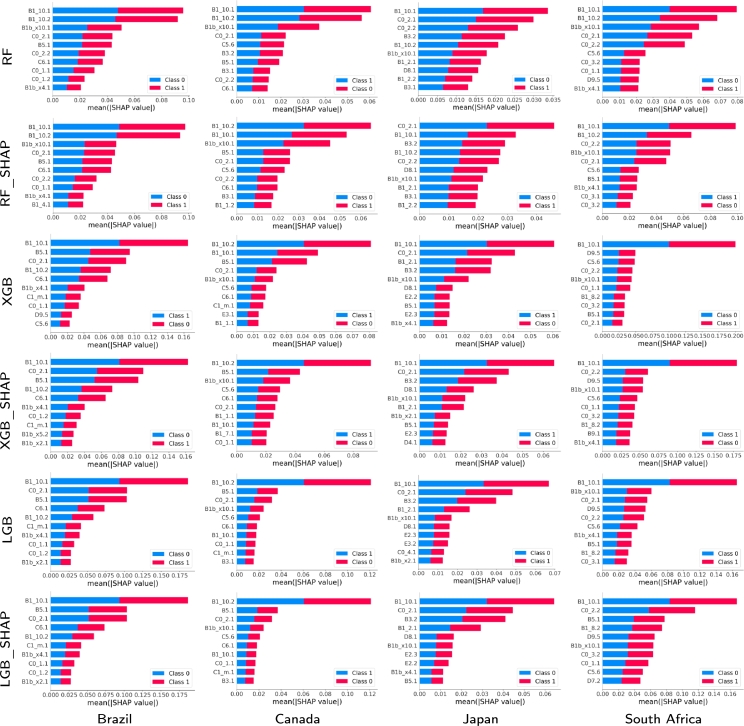
SHAP values and their impact on the detection output of the 10 most relevant features obtained by the proposed approach using different classification models for the four countries and for 2020.

**Figure 8:**
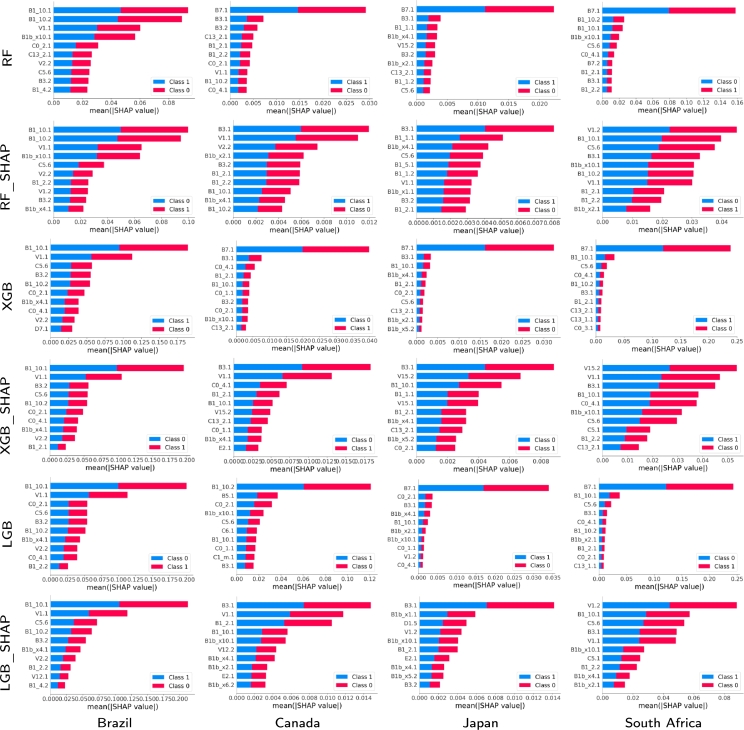
SHAP values and their impact on the detection output of the 10 most relevant features obtained by the proposed approach using different classification models for the four countries and for 2021.

We now proceed to analyze the explainability based on the results given by the different techniques, using (or not) the Shapley-based feature removal, to estimate positive cases, for the four countries and for 2020 and 2021 (see Figure 7, Figure 8). We will delve deeper into the model that gave the best results, both in its full version and with the Shapley-based feature reduction. In the analysis, we have considered that the variables with Shapley values greater than 0.05 are relevant to consider, as stipulated in the literature [20], [46].

• Year 2020: According to Fig. 7, in the case of RF the variable B1.10.1 is relevant in all cases (countries), while in other cases, the variable B1.10.2 is also relevant as C0.2.1 (it is the most relevant for Japan but with a low relevance value of less than 0.03). This makes sense because these variables refer to the loss of smell/taste (B1.10.1 and B1.10.2), and the fact of having gone to a market, store or pharmacy (C0.2.1). By 2020, the most widespread symptom was loss of smell and taste. B1.10.1, even in the other methods, had an even higher relevance value, reaching values greater than 0.1 in LGB and LGB.SHAP for Brazil and South Africa, and 0.08 in XGB and XGB.SHAP. Also, variable B1bx10.1, which means that a usual symptom was the loss of taste or smell, appears relevant in the case of South Africa for RF (close to 0.03). This is very much in agreement with what happened for the variant that prevailed in 2020.
  In general, for 2020, the most important variable in every model is the loss of smell or taste. Similarly to what was determined by [56], [57], this was the most representative symptom of COVID-19 with the first variant, the only one present in 2020. Along with the variables described in the previous paragraph, other variables somewhat relevant (in some cases, with Shapley values greater than 0.04) were B5.1 (spent time with COVID-19 infected people), C6.1 (Not spend time with someone outside your household) or C5.6 (Not going out for a week) to determine if an individual is COVID-19 positive.
  To continue with the explainability analysis, and as an example, we did our own ranking to determine the relevance of the features in general, according to the order using the Shapley value in which a feature appears in each technique for each country. For this ranking, the first 10 variables were considered according to the Shapley value for each technique in each country, and a 10 was assigned to the one with the highest Shapley value, 9 to the next, and so on. Then, at the end, the values obtained by each feature in each country-technique pair are added to obtain its position in our ranking.
  Table 6 lists only the first 10 variables according to that ranking. Each column contains the ranking of the ten most important features for each constructed model based on Shapley values. As the value of the cell increases, the importance of the feature increases, with one being the least important feature and ten being the most important. A zero value indicates that the variable is not on the list of the ten most important features. In the last column, you will find the sum of importance across the models. We can see that the variable B1.10.1 has a value much higher than the rest (218), and with C0.2.1 (192) they are very far from the rest. They are the same variables that we had determined before as the most relevant, which corroborates our previous explainability analysis. B1.10.1 has the highest value in almost all cases, and only in Canada, for some techniques, it has a low value (for example, XGB with 3). This happens less in C0.2.1, since its lowest value is 5 (also for XGB and Canada). The rest of the techniques have at least once a value of 0, which means that they are not among the first 10 variables according to the Shapley value in that technique and country.

**Table 6:**
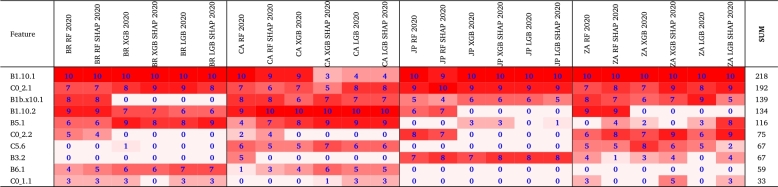
Ranking of the features based on the Shapley values for the entire set of countries for 2020.
  We also see some cases where the techniques in some countries only use a few of the best ranked variables according to our ranking. For example, the case of Brazil with XGB using Shapley, or Japan with XGB, which use only 5 of the first 10 variables of our ranking. That implies that they have other more relevant variables, in addition to those 5, to the first 10 established by our ranking (for example, V1.1 has the second largest Shapley value in Japan with XGB). We also see that there is no technique for any country that uses those 10 best ranked variables according to our criteria, only RF for Canada used 9 of them.
• Year 2021: This year was characterized by the appearance of several variants of COVID-19 (Delta, Omicron, etc.) and by the massive vaccination of the population against COVID-19. In this case, the variable B1.10.1 continued to appear as one of the most relevant variables, and in some cases B1.10.2. However, other variables that also appeared with great relevance were V1.1 and B7.1. These variables refer to whether the person has been vaccinated against COVID-19 (V1.1), and if in the last 4 weeks the person did any paid work (B7.1). Particularly, V1.1 is relevant in Brazil (despite being a country where some government instances promoted the denial of the positive effect of vaccination), and B7.1 is the most relevant on several occasions (for example, for RF and XGB in Canada, South Africa and Japan, although with low relevance values, of the order of 0.015). Other variables that appear with some relevance in some cases are B3.1 that deals with whether someone in the local community is known to have been ill with fever, cough, or difficulty breathing (for example, the most relevant for RF.SHAP in Canada and Japan, among others, but with a very low relevance (less than 0.004)), B15.2 which is if the person has had an appointment to receive a COVID-19 vaccine (the most relevant for XGB.SHAP in South Africa), and V1.2 which deals with whether to have a COVID-19 vaccine (the most relevant for XGB.SHAP in South Africa with 0.05).
  Variable B3.1 is the most relevant for all SHAP-based methods for Canada and Japan, which indicates whether anyone in the local community was known to have fever and cough or shortness of breath as one of the causes of seropositivity (rapid spread of the virus). Similarly, variable B7.1 is the most relevant for all methods without SHAP for Japan, Canada and South Africa (contagion from going to work, also linked to the rapid spread of the virus). Finally, V1.2 is the most relevant for all methods with SHAP in South Africa, indicating the fact of not having been vaccinated as one of the reasons for high seropositivity in that country. In the case of Brazil, the most relevant variables were again B1.10.1, B1.10.2 and V1.1. Also to note that C0.2.1 ceased to be relevant in 2021.
  For 2021, again, one of the most important features is the loss of smell or taste. However, with less relevance in some countries as it was in 2020 due to the COVID-19 variants during this year along with the vaccination. In addition, variables linked to facilitating the spread (such as going to work (B7.1) or if the person had acquaintances with symptoms (B3.1)) appear as reasons for seropositivity, or the case of not having been vaccinated yet (South Africa and V1.2). Thus, 2021 has some of the same important features as 2020 but the variables related to the vaccines and the rapid spread of the virus also play a key role.

#### 4.2.2 Explainability analysis using the range of features given by RF

**Figure 9:**
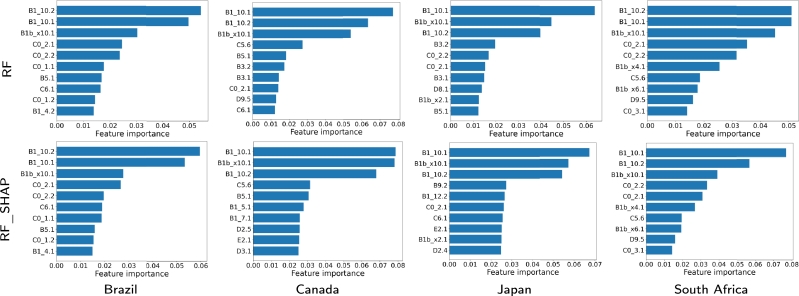
Feature importance of the 10 most relevant input variables obtained by classification models based on the random forest method for the four countries and for 2020.

**Figure 10:**
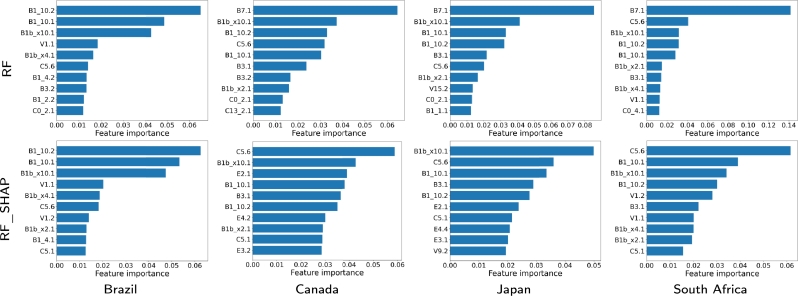
Feature importance of the 10 most relevant input variables obtained by classification models based on the random forest method for the four countries and for 2021.

In this part, we carry out an explainability analysis for RF because it is the technique that showed the best quality (see Figure 9, Figure 10). With RF, various measures of feature importance can be used for an explainability analysis. In this work, we have used MDA for being one of the best feature-importance measures according to the literature [41], [45].

• 2020: It is interesting to see that again, the most relevant variables are B1.10.1, B1.10.2, as well as B1bx10.1. The relevance order changes in some cases, as for RF (B1.10.1 is the variable that appears as relevant most frequently for RF using the Shapley values, and in this case is shared with B1.10.2). In general, the relevance values of the most relevant variables are always high, and occasionally very high (for example, B1.10.1 for Canada close to 0.8).
  Another aspect to note is how in some cases the most frequent variables change, as in the case of Japan with C0.2.1 (it was among the most relevant variables in the Shapley values and was no longer among the three most relevant in the RF ranking). Finally, B1.10.1 and B1.10.2 are always the most relevant, with values greater than 0.05 regardless of whether RF or RF.SHAP is used. This clearly indicates that the variables linked to loss of smell/taste are the fundamental ones for estimating seropositivity to COVID-19 in the case of RF and RF.SHAP.
• 2021: In the case of Brazil, the most relevant variables were once again B1.10.1, B1.10.2, with B1.10.2 now being more relevant. On the other hand, the variable V1.1 disappears from the relevant group (which makes sense, because Brazil was a country where the denial of the positive effect of vaccination was promoted from some of the government instances). Also, variable B7.1 continues to be the most relevant for all methods without SHAP for Japan, Canada and South Africa, being very decisive in Japan and Canada (values greater than 0.08, and the next with relevance values around 0.04). Another variable that is no longer relevant is B3.1, with two variables appearing as highly relevant, B1bx10.1 and C5.6, particularly for SHAP-based techniques. B1bx10.1 is relevant in Japan, which means that a common symptom was the loss of taste or smell; and C5.6 in South Africa and Canada, and it has to do with not having been in public during the last 7 days (self-care of people).

Note that the most important features selected for RF with the full set of input variables differ from those selected for RF.SHAP for each country and period. The difference is due to the fact that the feature selection method extracts the Shapley values while the feature rankings are computed using the MDA method. As can be seen in the explainability analysis, this approach provides information on additional features that contribute to active case detection.

## 5 Discussion

### 5.1 Performance quality of the machine-learning approaches

This study presents a machine-learning approach to detecting COVID-19-active cases based on three classification models: random forest (RF), light gradient boosting (LGB), and extreme gradient boosting (XGB). More precisely, the proposed detection approach predicts active cases using the entire set of variables collected from the UMD-CTIS questionnaires. These questionnaires record a wide range of individual features such as gender, age group, vaccination acceptance, and isolation measures. In addition, we introduce a feature-reduction approach that uses the RFE strategy to train the classification model. A key objective of the RFE algorithm is to identify and keep relevant features based on Shapley values without compromising detection accuracy. It is pertinent to mention that the proposed method is evaluated on UMD-CTIS data extracted from four countries: Brazil, Canada, Japan, and South Africa, and two periods: 2020 and 2021. Specifically, we consider experiments with at least one symptom reported within the past 24 hours and a test result within the past 14 days. Extracted datasets may contain biases, limitations, and missing values. In countries where demographic information can be a significant factor in detecting active cases, such as isolated communities and islands, the data may be affected by biases due to the homogenization of the population. In addition, biases can arise from assuming that all populations have Internet access uniformly. To reduce biases, we randomly select a limited set of experiments as the training set to optimize the classification models.

The proposed approach has shown competitive performance for the four countries for 2020 and 2021. In particular, the feature selection stage removes a large number of irrelevant variables with a negligible impact on classification accuracy. According to different quality metrics (such as $F_{1}$-score, sensitivity, specificity, precision, and AUC), RF and RF SHAP models exhibit the most accurate detection performances across the four countries for 2020 and 2021. We also compare the performance of the developed technique to those yielded by previously reported approaches based on surveys. The proposed detection methodology outperforms the state-of-the-art methods for the four countries in terms of $F_{1}$-score. The RF-based approach obtained the highest results, regardless of whether or not feature selection was used. In the final step, we use the developed detection approach to construct the normalized daily-case curve for the four countries between January 1, 2021, and June 25, 2022, to observe pandemic trends. In comparison with official records, these estimated curves provide consistent tracking of pandemic evolution. Therefore, taking into account both the proposed detection approaches and the massive amount of data provided by the UMD-CTIS questionnaires, we can reliably track pandemic indicator trends in a similar way to that provided by public healthcare systems.

### 5.2 Explainability analysis

Regarding the explainability analysis, the variables that appear most frequently are the loss of smell or taste (B1.10.1/B1.10.2), regardless of the year, country, the technique used for the explainability analysis, the prediction technique, or dataset reduced or not by the Shapley method. Other variables appear in some specific cases (countries, forecasting techniques, etc.). For example, using the Shapley method for explainability analysis in 2020, the variable C0.2.1 (the fact of having gone to a market, store or pharmacy) is relevant in some countries. Also, in 2021, the variable V1.1 is relevant in Brazil (whether the person has been vaccinated against COVID-19), the variable B7.1 is relevant in some countries (if in the last 4 weeks the person did any paid work) for the cases without data reduction, and B3.1 (it indicates whether anyone in the local community was known to have fever and cough) for cases with data reduction using the Shapley method. The same happens using the RF ranking for the explainability analysis: new variables appear (such as C5.6) or some existing ones disappear (such as C0.2.1 and B3.1).

In any case, attribute-based explainability analysis shows the relevant variables for decision makers to detect seropositivity very quickly. This is valid both for the case of using the ranking given by RF or the Shapley values. However, it is important to highlight that although they present common variables, between the two techniques there are some differences between those that are considered to be more relevant. For example, V1.1 appears as relevant in the Shapley method for Brazil and disappears in the RF ranking, which makes more sense because in Brazil, the vaccination campaign did not have a strong support from the government.

Thus, RF seems sufficient to achieve good results and explain the results obtained (explainability analysis). But although explainability is aimed at the understanding by experts and non-experts, there are no designs or formal evaluations on the human usability of the methods analyzed in this work. This is pending work, which goes beyond simple representations of explanations. At the same time, the analysis of the variables by classes has rarely been carried out (in the case of Shapley, the values by class are similar/symmetrical), which opens up a space for research for the development of techniques that allow analyzing the possibility of explainability by classes (which characteristics/variables are relevant for each class).

Now, in this last part, we define a variable pattern by combining the results of the two explainability analysis techniques (Shapley value and feature importance) for each year and country/year. According to the explainability analysis results, we build symptomatic patterns using the relevance of the features in both techniques. Thus, the relevant features for each year are:

• **2020:** Loss of smell and taste in the last 24 h (B1.10.1)
• **2021:** Loss of smell and taste in the last 24 h (B1.10.1); and COVID-19 vaccination in the last 24 h (V1.1).

For all countries in 2020, the most frequent variable is loss of smell and taste in the last 24 hours (variable B1.10.1). Additionally, vaccination campaigns in 2021 become a relevant factor in the positive prediction of COVID-19. For country/year, the relevant features are:

• **Brazil 2020:** Loss of smell and taste in the last 24 h (B1.10.1); Loss of smell and taste after 24 hours (B1.x10.1); Have you gone to a market, grocery store, or pharmacy in the last 24 hours (C02.1), Have you spent time with any of these people in the last 7 days? (B5.1).
• **Brazil 2021:** Loss of smell and taste in the last 24 h (B1.10.1); Loss of smell and taste after 24 hours (B1.x10.1), Have Fatigue? (B1b.x4.1).
• **Canada 2020:** Loss of smell and taste in the last 24 h (B1.10.1); Loss of smell and taste after 24 hours (B1.x10.1); Have you spent time with any of these people in the last 7 days? (B5.1); In the last 7 days, have you not been in public? (C5.6).
• **Canada 2021:** Loss of smell and taste in the last 24 h (B1.10.1); Loss of smell and taste after 24 hours (B1.x10.1); Do you personally know anyone in your local community who is sick with a fever and either a cough or difficulty breathing? (B3.1).
• **Japan 2020:** Loss of smell and taste after 24 hours (B1.x10.1); Loss of smell and taste in the last 24 h (B1.10.1); have you gone to a market, grocery store, or pharmacy in the last 24 hours (C02.1).
• **Japan 2021:** Do you have a cough? (B1b.x2.1); Do you personally know anyone in your local community who is sick with a fever and either a cough or difficulty breathing? (B3.1).
• **South Africa 2020:** Loss of smell and taste after 24 hours (B1.x10.1); Loss of smell taste in the last 24 h (B1.10.1); Have you gone to a market, grocery store, or pharmacy in the last 24 hours (C02.1); In the last 7 days, have you not been in public? (C5.6).
• **South Africa 2021:** Loss of smell and taste after 24 hours (B1.x10.1); Loss of smell and taste in the last 24 h (B1.10.1); Do you personally know anyone in your local community who is sick with a fever and either a cough or difficulty breathing? (B3.1); In the last 7 days, have you not been in public? (C5.6); Have you had a COVID-19 vaccination? (V1.1).

As can be seen above, the most relevant variables are loss of smell and taste after 24 hours (B1.x10.1) and loss of smell and taste in the last 24 hours (B1.10.1), which appear for all countries. The rest of the variables are very specific to each country. During the pandemic, each country experienced different health, geographical, or economic conditions (for example, the bad South Africa's vaccination campaign in 2021 turned off the variable V1.1; or the fact that the Brazilian authorities denied the pandemic during 2020 affected the variables “Have you been to the market, grocery store, or pharmacy in the last 24 hours?” (C02.1) or “Have you spent time with any of these people within the last week?” (B5.1)). In summary, as we have mentioned before, the only variable that appears in all explainability analyses, regardless of explainability technique, or machine learning method used, is loss of smell and taste after 24 hours.

## Ethical declaration

The Ethics Board (IRB) of IMDEA Networks Institute gave ethical approval for this work on 2021/07/05. IMDEA Networks has signed Data Use Agreements with Facebook, Carnegie Mellon University (CMU) and the University of Maryland (UMD) to access their data, specifically UMD project 1587016-3 entitled C-SPEC: Symptom Survey: COVID-19 and CMU project STUDY2020_00000162 entitled ILI Community-Surveillance Study. The data used in this study was collected by the University of Maryland through The University of Maryland Social Data Science Center Global COVID-19 Trends and Impact Survey in partnership with Facebook. Informed consent has been obtained from all participants in this survey by this institution. All the methods in this study have been carried out in accordance with relevant of ethics and privacy guidelines and regulations.

## CRediT authorship contribution statement

**Jesús Rufino:** Conceptualization, Data curation, Formal analysis, Investigation, Methodology, Software, Writing – original draft. **Juan Marcos Ramírez:** Formal analysis, Investigation, Validation, Visualization, Writing – original draft, Writing – review & editing, Methodology. **Jose Aguilar:** Formal analysis, Investigation, Methodology, Supervision, Visualization, Writing – original draft, Writing – review & editing. **Carlos Baquero:** Conceptualization, Formal analysis, Funding acquisition, Investigation, Methodology, Resources, Supervision, Writing – review & editing. **Jaya Champati:** Conceptualization, Formal analysis, Investigation, Methodology, Supervision, Writing – review & editing. **Davide Frey:** Conceptualization, Formal analysis, Investigation, Methodology, Supervision, Writing – review & editing. **Rosa Elvira Lillo:** Conceptualization, Formal analysis, Funding acquisition, Investigation, Methodology, Project administration, Resources, Supervision, Writing – original draft, Writing – review & editing. **Antonio Fernández-Anta:** Conceptualization, Data curation, Formal analysis, Funding acquisition, Investigation, Methodology, Project administration, Resources, Supervision, Writing – original draft, Writing – review & editing.

## Declaration of Competing Interest

The authors declare that they have no known competing financial interests or personal relationships that could have appeared to influence the work reported in this paper.

## Data Availability

The data presented in this paper (in aggregated form) and the programs used to process it will be openly accessible at https://github.com/GCGImdea/coronasurveys/. The microdata of the CTIS survey from which the aggregated data was obtained cannot be shared, as per the Data Use Agreements signed with Facebook, Carnegie Mellon University (CMU) and the University of Maryland (UMD). Thus, the authors do not have permission to share the microdata of the CTIS survey.

## Appendix A Supplementary material

MMC Feature Selection for an Explainability Analysis in Detection of COVID-19 Active Cases in Countries from Facebook User-Based Online Surveys. MMC

## References

[1] Astley C.M., Tuli G., Mc Cord K.A., Cohn E.L., Rader B., Varrelman T.J., Chiu S.L., Deng X., Stewart K., Farag T.H. (2021). Global monitoring of the impact of the Covid-19 pandemic through online surveys sampled from the facebook user base. Proc. Natl. Acad. Sci..

[2] Akinbami L.J., Petersen L.R., Sami S., Vuong N., Lukacs S.L., Mackey L., Atas J., LaFleur B.J. (2021). Coronavirus disease 2019 symptoms and severe acute respiratory syndrome coronavirus 2 antibody positivity in a large survey of first responders and healthcare personnel, May-July 2020. Clin. Infect. Dis..

[3] M. Klompas, Coronavirus disease 2019 (Covid-19): protecting hospitals from the invisible, 2020. 10.7326/M20-0751 PMC7081175 32160299

[4] Zoabi Y., Deri-Rozov S., Shomron N. (2021). Machine learning-based prediction of Covid-19 diagnosis based on symptoms. npj Digit. Med..

[5] Smith D.S., Richey E.A., Brunetto W.L. (2020). A symptom-based rule for diagnosis of Covid-19. SN Compr. Clin. Med..

[6] Menni C., Valdes A.M., Freidin M.B., Sudre C.H., Nguyen L.H., Drew D.A., Ganesh S., Varsavsky T., Cardoso M.J., Moustafa J.S.E.-S. (2020). Real-time tracking of self-reported symptoms to predict potential COVID-19. Nat. Med..

[7] Chan A.T., Brownstein J.S. (2020). Putting the public back in public health—surveying symptoms of Covid-19. N. Engl. J. Med..

[8] Allen W.E., Altae-Tran H., Briggs J., Jin X., McGee G., Shi A., Raghavan R., Kamariza M., Nova N., Pereta A. (2020). Population-scale longitudinal mapping of Covid-19 symptoms, behaviour and testing. Nat. Hum. Behav..

[9] Roland L.T., Gurrola J.G., Loftus P.A., Cheung S.W., Chang J.L. (2020). International Forum of Allergy & Rhinology.

[10] Rufino J., Ramírez J.M., Aguilar J., Baquero C., Champati J., Frey D., Lillo R.E., Fernández-Anta A. (2023). Consistent comparison of symptom-based methods for Covid-19 infection detection. Int. J. Med. Inform..

[11] Coronavirus disease 2019 (COVID-19) 2020 interim case definition, Approved April 5, 2020, National Notifiable Diseases Surveillance System (NNDSS), 2020.

[12] World Health Organization (2020). Coronavirus disease (COVID-19) Q&A. https://www.who.int/news-room/q-a-detail/coronavirus-disease-covid-19.

[13] J. Álvarez, C. Baquero, E. Cabana, J.P. Champati, A.F. Anta, D. Frey, A. Garcia-Agundez, C. Georgiou, M. Goessens, H. Hernández, R. Lillo, R. Menezes, R. Moreno, N. Nicolaou, O. Ojo, A. Ortega, E. Rausell, J. Rufino, E. Stavrakis, G. Jeevan, C. Glorioso, Estimating active cases of COVID-19, medRxiv, 2021.

[14] Pérez-Gómez B., Pastor-Barriuso R., Pérez-Olmeda M., Hernán M.A., Oteo-Iglesias J., de Larrea N.F., Fernández-García A., Martín M., Fernández-Navarro P., Cruz I. (2021). Ene-covid nationwide serosurvey served to characterize asymptomatic infections and to develop a symptom-based risk score to predict Covid-19. J. Clin. Epidemiol..

[15] Salomon J.A., Reinhart A., Bilinski A., Chua E.J., La Motte-Kerr W., Rönn M.M., Reitsma M.B., Morris K.A., LaRocca S., Farag T.H. (2021). The US Covid-19 trends and impact survey: continuous real-time measurement of Covid-19 symptoms, risks, protective behaviors, testing, and vaccination. Proc. Natl. Acad. Sci..

[16] S. Shoer, T. Karady, A. Keshet, S. Shilo, H. Rossman, A. Gavrieli, T. Meir, A. Lavon, D. Kolobkov, I. Kalka, et al., Who should we test for Covid-19? A triage model built from national symptom surveys, medRxiv, 2020. 10.1016/j.medj.2020.10.002 PMC7547576 33073258

[17] Mika J., Tobiasz J., Zyla J., Papiez A., Bach M., Werner A., Kozielski M., Kania M., Gruca A., Piotrowski D. (2021). Symptom-based early-stage differentiation between sars-cov-2 versus other respiratory tract infections—Upper Silesia pilot study. Sci. Rep..

[18] Bhattacharya A., Ranjan P., Kumar A., Brijwal M., Pandey R.M., Mahishi N., Baitha U., Pandey S., Mittal A., Wig N. (2021). Development and validation of a clinical symptom-based scoring system for diagnostic evaluation of Covid-19 patients presenting to outpatient department in a pandemic situation. Cureus.

[19] Kreuter F., Barkay N., Bilinski A., Bradford A., Chiu S., Eliat R., Fan J., Galili T., Haimovich D., Kim B. (2020). Survey Research Methods.

[20] Chen J., Yuan S., Lv D., Xiang Y. (2021). A novel self-learning feature selection approach based on feature attributions. Expert Syst. Appl..

[21] Holzinger A., Langs G., Denk H., Zatloukal K., Müller H. (2019). Causability and explainability of artificial intelligence in medicine. Interdiscip. Rev. Data Min. Knowl. Discov..

[22] Nyrup R., Robinson D. (2022). Explanatory pragmatism: a context-sensitive framework for explainable medical ai. Ethics Inf. Technol..

[23] Gabbay F., Bar-Lev S., Montano O., Hadad N. (2021). A lime-based explainable machine learning model for predicting the severity level of Covid-19 diagnosed patients. Appl. Sci..

[24] Girardi I., Vagenas P., Arcos-Diaz D., Bessa I., Bu-Sser A., Furlan L., Furlan R., Gatti M., Giovannini A., Hoeven E., Marchiori C. (2021). AMIA Annu. Symp. Proc..

[25] Novak J., Maljur T., Drenska K., Chen J.Y.C., Fragomeni G., Degen H., Ntoa S. (2022). HCI International 2022 – Late Breaking Papers: Interacting with eXtended Reality and Artificial Intelligence.

[26] Excoffier J.-B., Salaün-Penquer N., Ortala M., Raphaël-Rousseau M., Chouaid C., Jung C. (2022). Analysis of Covid-19 inpatients in France during first lockdown of 2020 using explainability methods. Med. Biol. Eng. Comput..

[27] Yu Z., Sohail A., Nofal T.A., Tavares J.M.R. (2022). Explainability of neural network clustering in interpreting the Covid-19 emergency data. Fractals.

[28] Phongchit N., Taeprasartsit P. (2021). 2021 25th International Computer Science and Engineering Conference (ICSEC).

[29] Aldhahi W., Sull S. (2023). Uncertain-cam: uncertainty-based ensemble machine voting for improved Covid-19 cxr classification and explainability. Diagnostics.

[30] Ali S., Hussain A., Bhattacharjee S., Athar Abdullah A., Kim H.-C. (2022). Detection of Covid-19 in x-ray images using densely connected squeeze convolutional neural network (dcscnn): focusing on interpretability and explainability of the black box model. Sensors.

[31] Saxena P., Singh S.K., Tiwary G., Mittal Y., Jain I. (2022). 2022 IEEE International Conference on Distributed Computing and Electrical Circuits and Electronics (ICDCECE).

[32] Li M., Li X., Jiang Y., Zhang J., Luo H., Yin S. (2022). Explainable multi-instance and multi-task learning for Covid-19 diagnosis and lesion segmentation in ct images. Knowl.-Based Syst..

[33] Kathamuthu N.D., Subramaniam S., Le Q.H., Muthusamy S., Panchal H., Sundararajan S.C.M., Alrubaie A.J., Zahra M.M.A. (2023). A deep transfer learning-based convolution neural network model for Covid-19 detection using computed tomography scan images for medical applications. Adv. Eng. Softw..

[34] Deeb A., Debow A., Mansour S., Shkodyrev V. (2023). Covid-19 diagnosis with deep learning: adjacent-pooling ctscan-Covid-19 classifier based on resnet and cbam. Biomed. Signal Process. Control.

[35] Ullah Z., Usman M., Gwak J. (2023). Mtss-aae: multi-task semi-supervised adversarial autoencoding for Covid-19 detection based on chest x-ray images. Expert Syst. Appl..

[36] Ershadi M., Rise Z. (2023). Fusing clinical and image data for detecting the severity level of hospitalized symptomatic Covid-19 patients using hierarchical model. Res. Biomed. Eng..

[37] Arabameri A., Pal S.C., Rezaie F., Chakrabortty R., Saha A., Blaschke T., Napoli M.D., Ghorbanzadeh O., Ngo P.T.T. (2022). Decision tree based ensemble machine learning approaches for landslide susceptibility mapping. Geocarto Int..

[38] Yasir M., Karim A.M., Malik S.K., Bajaffer A.A., Azhar E.I. (2022). Application of decision-tree-based machine learning algorithms for prediction of antimicrobial resistance. Antibiotics.

[39] Liew X.Y., Hameed N., Clos J. (2021). An investigation of xgboost-based algorithm for breast cancer classification. Mach. Learn. Appl..

[40] Ramirez J.M., Torre J.I.M., Arguello H. (2021). Feature fusion via dual-resolution compressive measurement matrix analysis for spectral image classification. Signal Process. Image Commun..

[41] Birant K.U. (2022). Multi-view rank-based random forest: a new algorithm for prediction in esports. Expert Syst..

[42] Delgado-Panadero A., Hernández-Lorca B., García-Ordás M.T., Benítez-Andrades J.A. (2022). Implementing local-explainability in gradient boosting trees: feature contribution. Inf. Sci..

[43] Burkart N., Huber M.F. (2021). A survey on the explainability of supervised machine learning. J. Artif. Intell. Res..

[44] Biran O., Cotton C. (2017). IJCAI-17 Workshop on Explainable AI (XAI).

[45] Alam M.Z., Rahman M.S., Rahman M.S. (2019). A random forest based predictor for medical data classification using feature ranking. Inform. Med. Unlock..

[46] Messalas A., Kanellopoulos Y., Makris C. (2019). 2019 10th International Conference on Information, Intelligence, Systems and Applications (IISA).

[47] James G., Witten D., Hastie T., Tibshirani R. (2013).

[48] Jović A., Brkić K., Bogunović N. (2015). 2015 38th International Convention on Information and Communication Technology, Electronics and Microelectronics (MIPRO).

[49] Kohavi R., John G.H. (1997). Wrappers for feature subset selection. Artif. Intell..

[50] Guyon I., Makhoul J., Schwartz R., Vapnik V. (1998). What size test set gives good error rate estimates?. IEEE Trans. Pattern Anal. Mach. Intell..

[51] Riley R.D., Ensor J., Snell K.I., Harrell F.E., Martin G.P., Reitsma J.B., Moons K.G., Collins G., Van Smeden M. (2020). Calculating the sample size required for developing a clinical prediction model. BMJ, Br. Med. J..

[52] Japkowicz N., Shah M. (2011).

[53] Demšar J. (2006). Statistical comparisons of classifiers over multiple data sets. J. Mach. Learn. Res..

[54] Rufino J., Baquero C., Frey D., Glorioso C.A., Ortega A., Reščič N., Roberts J.C., Lillo R.E., Menezes R., Champati J.P. (2023). Using survey data to estimate the impact of the omicron variant on vaccine efficacy against Covid-19 infection. Sci. Rep..

[55] Baquero C., Casari P., Fernandez Anta A., García-García A., Frey D., Garcia-Agundez A., Georgiou C., Girault B., Ortega A., Goessens M. (2021). The coronasurveys system for Covid-19 incidence data collection and processing. Front. Comput. Sci..

[56] Mullol J., Alobid I., Mariño-Sánchez F., Izquierdo-Domínguez A., Marin C., Klimek L., Wang D., Liu Z. (2020). The loss of smell and taste in the Covid-19 outbreak: a tale of many countries. Curr. Allergy Asthma Rep..

[57] Hannum M., Koch R., Ramirez V., Marks S., Toskala A., Herriman R., Lin C., Joseph P., D.R. R. (2022). Taste loss as a distinct symptom of Covid-19: a systematic review and meta-analysis. Chem. Senses.

