# Supplementary material for: Performance and explainability of feature selection-boosted tree-based classifiers for COVID-19 detection
Source: Heliyon. 2023 Dec 7;10(1):e23219. doi: 10.1016/j.heliyon.2023.e23219 (PMC10758803; doi:10.1016/j.heliyon.2023.e23219)
Supplement: MMC — Feature Selection for an Explainability Analysis in Detection of COVID-19 Active Cases in Countries from Facebook User-Based Online Surveys. [file mmc1.pdf]

Supplemental Materials: Feature Selection for an Explainability Analysis in Detection of COVID-19 Active Cases in Countries from Facebook User-Based Online Surveys

1. Questions of the survey

- **B1 In the last 24 hours, have you had any of the following?** Fever (B1_1), Cough (B1_2), Difficulty breathing (B1_3), Fatigue (B1_4), Stuffy or runny nose (B1_5), Aches or muscle pain (B1_6), Sore throat (B1_7), Chest pain (B1_8), Nausea (B1_9), Loss of smell or taste (B1_10), Headache (B1_12), Chills (B1_13).
- **B1b Are any of these symptoms unusual for you?** Fever (B1b_x1), Cough (B1b_x2), Difficulty breathing (B1b_x3), Fatigue (Bb1_x4), Stuffy or runny nose (B1b_x5), Aches or muscle pain (B1b_x6), Sore throat (B1b_x7), Chest pain (B1b_x8), Nausea (B1b_x9), Loss of smell or taste (B1b_x10), Headache (B1b_x12), Chills (B1b_x13).
- **B3 Do you personally know anyone in your local community who is sick with a fever and either a cough or difficulty breathing?** Yes(1), No(2).
- **B5 Have you spent time with any of these people in the last 7 days?** Yes(1), No(2)[1]
- **B6 Have you ever been tested for coronavirus (COVID-19)?** Yes(1), No(2)[1]
- **B7 Have you been tested for COVID-19 in the past 14 days?** Yes(1), No(2).
- **B8 Did your most recent test find that you had COVID-19?** Yes(1), No(2), I don't know(3).
- **B9 Did you have to pay anything out-of-pocket for this test?** Yes(1), No(2), I don't know(3).[1]
- **B10 Have you or your household had to reduce spending on things you need (such as food,housing, or medication) because of the cost you paid to get the coronavirus (COVID-19) test?** Yes(1), No(2), I don't know(3).[1]
- **B10 Have you or your household had to reduce spending on things you need (such as food, housing, or medication) because of the cost you paid to get the coronavirus (COVID-19) test?** Yes(1), No(2), I don't know(3).[1]
- **B12_1 Do any of the following reasons describe why you haven't been tested for coronavirus (COVID-19) in the last 14 days? I tried to get a test but was not able to get one** Yes(1), No(2).[1]
- **B12_2 Do any of the following reasons describe why you haven't been tested for coronavirus (COVID-19) in the last 14 days? I don't know where to go** Yes(1), No(2).[1]
- **B12_3 Do any of the following reasons describe why you haven't been tested for coronavirus (COVID-19) in the last 14 days? I can't afford the cost of the test** Yes(1), No(2).[1]
- **B12_4 Do any of the following reasons describe why you haven't been tested for coronavirus (COVID-19) in the last 14 days? I don't have time to get tested** Yes(1), No(2).[1]
- **B12_5 Do any of the following reasons describe why you haven't been tested for coronavirus (COVID-19) in the last 14 days? I am unable to travel to a testing location (including because of transportation cost, safety, or physical limitations)** Yes(1), No(2).[1]
- **B12_6 Do any of the following reasons describe why you haven't been tested for coronavirus (COVID-19) in the last 14 days? I am worried about bad things happening to me or my family (including discrimination, government policies, and social stigma)** Yes(1), No(2).[1]
- **B13_1 In the last 30 days, was there any time when you needed any of the following health services or products but could not get it? Emergency transportation services or emergency rescue** Yes(1), No(2).[1]

[1]Only for 2020

- **B13_2 In the last 30 days, was there any time when you needed any of the following health services or products but could not get it? Medical care with overnight stay in any type of facility** Yes(1), No(2).[1]
- **B13_3 In the last 30 days, was there any time when you needed any of the following health services or products but could not get it? Medical or dental care or treatment without an overnight stay** Yes(1), No(2).[1]
- **B13_4 In the last 30 days, was there any time when you needed any of the following health services or products but could not get it? Preventive health services (including immunization/vaccination, family planning, prenatal/postnatal care, routine check-up services)** Yes(1), No(2).[1]
- **B13_5 In the last 30 days, was there any time when you needed any of the following health services or products but could not get it? Medication** Yes(1), No(2).[1]
- **B13_6 In the last 30 days, was there any time when you needed any of the following health services or products but could not get it? Mask, medical gloves, or other protective equipment** Yes(1), No(2).[1]
- **B13_7 In the last 30 days, was there any time when you needed any of the following health services or products but could not get it? Eyeglasses, hearing aid, crutches, band-aids/plasters, thermometer, or any other health product** Yes(1), No(2).[1]
- **B14_1 In the last 30 days, were you unable to get needed treatment, services, medicine, or medical products for any of the following reasons? I didn't know where to go** Yes(1), No(2).[1]
- **B14_2 In the last 30 days, were you unable to get needed treatment, services, medicine, or medical products for any of the following reasons? I couldn't afford the treatment, service, or product** Yes(1), No(2).[1]
- **B14_3 In the last 30 days, were you unable to get needed treatment, services, medicine, or medical products for any of the following reasons? I was unable to travel to the health care provider (including because of transportation cost, safety, or physical limitations)** Yes(1), No(2).[1]
- **B14_4 In the last 30 days, were you unable to get needed treatment, services, medicine, or medical products for any of the following reasons? I was afraid of being infected at the health care provider** Yes(1), No(2).[1]
- **B14_5 In the last 30 days, were you unable to get needed treatment, services, medicine, or medical products for any of the following reasons?The treatment, service, or product was not available** Yes(1), No(2).[1]
- **C0_1 In the last 24 hours, have you done any of the following? Gone to work outside the place where you are currently staying** Yes(1), No(2)
- **C0_2 In the last 24 hours, have you done any of the following? Gone to a market, grocery store, or pharmacy** Yes(1), No(2)
- **C0_3 In the last 24 hours, have you done any of the following? Gone to a restaurant, cafe, or shopping center** Yes(1), No(2)
- **C0_4 In the last 24 hours, have you done any of the following? Spent time with someone who isn't currently staying with you** Yes(1), No(2)
- **C0_5 In the last 24 hours, have you done any of the following? Attended a public event with more than 10 people** Yes(1), No(2)
- **C0_6 In the last 24 hours, have you done any of the following? Used public transit** Yes(1), No(2)
- **C1_m In the last 24 hours, have you had direct contact with anyone who is not staying with you? Direct contact means spending longer than one minute within two meters of someone or touching, including shaking hands, hugging, or kissing.** Yes(1), No(2)[1]
- **C2 How many people, who are not staying with you, have you had this kind of direct contact with in the last 24 hours?** 1-4 people(1), 5-9 people(2), 10-19 people (3), 20 or more(4).[1]

- **C3 Do you have access to soap and water for washing your hands at the place where you are currently staying?** Yes(1), No(2)[1]
- **C5 In the last 7 days, how often did you wear a mask when in public?** All of the time(1), Most of the time(2), Some of the time(3), A little of the time(4), None of the time(5), I have not been in public during the last 7 days(6)
- **C6 In the last 7 days, how many days have you spent time with people who aren't staying with you?** 0 days(1), 1 day(2), 2-4 days(3),5-7 days(4).[1]
- **C7 In the last 24 hours, about how many times have you washed your hands with soap and water or used hand sanitizer?** 0 times (1), 1-2 times (2), 3-6 times (3),7 or more times (4).[1]
- **C8 Do you have access to soap and water for washing your hands at the place where you are currently staying?**Yes (1), No (2)[1]
- **C13a During which activities in the past 24 hours did you wear a mask? Please select all that apply.** Gone to work or school indoors, outside the place where you are currently staying (1), Gone to an indoor market, grocery store, or pharmacy (2), Had a drink or meal indoors at a bar, restaurant, or cafe (3), Spent time indoors with someone who isn't currently staying with you (4), Attended an indoor event with more than 10 people (5),Used public transit (6). [2]
- **D1 During the last 7 days, how often did you feel so nervous that nothing could calm you down?** All of the time(1), Most of the time(2), Some of the time(3), A little of the time(4), None of the time(5).
- **D2 During the last 7 days, how often did you feel so depressed that nothing could cheer you up?** All of the time(1), Most of the time(2), Some of the time(3), A little of the time(4), None of the time(5).
- **D3 How worried are you that you or someone in your immediate family might become seriously ill from coronavirus (COVID-19)?** Very worried(1), Somewhat worried(2), Not too worried(3), Not worried at all(4).[1]
- **D4 How worried are you about having enough to eat in the next week?** Very worried(1), Somewhat worried(2), Not too worried(3), Not worried at all(4).
- **D5 How worried are you about your household's finances in the next month?** Very worried(1), Somewhat worried(2), Not too worried(3), Not worried at all(4).
- **D7 In the past 4 weeks, did you do any work for pay? By work for pay, we mean any kind of business, farming, or other activity to earn money, even if only for one hour.** Yes (1), No(2).
- **D8 Before February 2020, were you working for pay, or doing any kind of business, farming, or other activity to earn money?** Yes(1), No(2)[1]
- **D9 Why did you stop working?** My employer closed for coronavirus-related reasons (1), My employer closed for another reason (2), I was laid off or furloughed (3), I am a seasonal worker (4), I was ill or quarantined (5), I needed to care for someone (6), Other (7)[1]
- **D10 What is the main activity of the business or organization in which you work?**Agriculture (1),Buying and selling (2), Construction (3),Education (4),Electricity / water / gas / waste (5),Financial / insurance / real estate services (6), Health (7), Manufacturing (8), Mining (9), Personal services (10), Professional / scientific / technical activities (11), Public administration (12), Tourism (13), Transportation (14), Other (15).
- **E2 Which of these best describes the area where you are currently staying?** City(1), Town(2), Village or rural area(3).
- **E3 What is your gender?** Male(1), Female(2), Other(3), Prefer not to answer(4)

[2]Only for 2021

- **E4 What is your age?** 18-24 years(1), 25-34 years(2), 35-44 years(3), 45-54 years(4), 55-64 years(5), 65-74 years(6), 75 years or older(7).
- **V1 Have you had a COVID-19 vaccination?**Yes (1), No (2),I don't know (3)[2]
- **V2 How many COVID-19 vaccinations have you received?**1 vaccination or dose (1), 2 vaccinations or doses (2),I don't know (3)[2]
- **V3 If a vaccine to prevent COVID-19 were offered to you today, would you choose to get vaccinated?** Yes, definitely (1), Yes, probably (2), No, probably not (3), No, definitely not (4)[2]
- **V5a Which of the following, if any, are reasons that you definitely wouldn't choose to get a COVID-19 vaccine? Please select all that apply.** I am concerned about possible side effects of a COVID-19 vaccine (1), I don't know if a COVID-19 vaccine will work (2), I don't believe I need a COVID-19 vaccine (3),I don't like vaccines (4), I plan to wait and see if it is safe and may get it later (5), I think other people need it more than I do right now (6), I am concerned about the cost of a COVID-19 vaccine (7), It is against my religious beliefs (8), I don't trust the government (10), Other (9)[2]
- **V5b Which of the following, if any, are reasons that you probably wouldn't choose to get a COVID-19 vaccine? Please select all that apply.** I am concerned about possible side effects of a COVID-19 vaccine (1), I don't know if a COVID-19 vaccine will work (2), I don't believe I need a COVID-19 vaccine (3), I don't like vaccines (4), I plan to wait and see if it is safe and may get it later (5),I think other people need it more than I do right now (6), I am concerned about the cost of a COVID-19 vaccine (7), It is against my religious beliefs (8), I don't trust the government (10), Other (9)[2]
- **V5c Which of the following, if any, are reasons that you probably wouldn't choose to get a COVID-19 vaccine? Please select all that apply.** I am concerned about possible side effects of a COVID-19 vaccine (1), I don't know if a COVID-19 vaccine will work (2), I don't believe I need a COVID-19 vaccine (3), I don't like vaccines (4), I plan to wait and see if it is safe and may get it later (5),I think other people need it more than I do right now (6), I am concerned about the cost of a COVID-19 vaccine (7), It is against my religious beliefs (8), I don't trust the government (10), Other (9).[2]
- **V6 Why don't you believe that you need a COVID-19 vaccine? Please select all that apply.**I already had COVID-19 (1), I do not spend time with any high-risk people (2), I am not a member of a high-risk group (3), I plan to use masks or other precautions instead (4), I don't believe COVID-19 is a serious illness (5), I don't think vaccines are beneficial (6), Other (7).[2]
- **V10 Have you ever been told by a doctor, nurse, or other health professional that you have any of the following medical conditions? Please select all that apply.** Asthma (1), Chronic lung disease such as COPD, chronic bronchitis, or emphysema (2), Cancer (3), Diabetes (4), High blood pressure (5), Kidney disease (6), Weakened or compromised immune system (7), Heart attack, heart disease, or other heart condition (8), Obesity (9), one of these (10).[2]
- **V15 Do you have an appointment to receive a COVID-19 vaccine?** Yes (1),No (2)[2]
- **V16 Have you tried to get an appointment to receive a COVID-19 vaccine?** Yes (1),No (2)[2]

2. Results

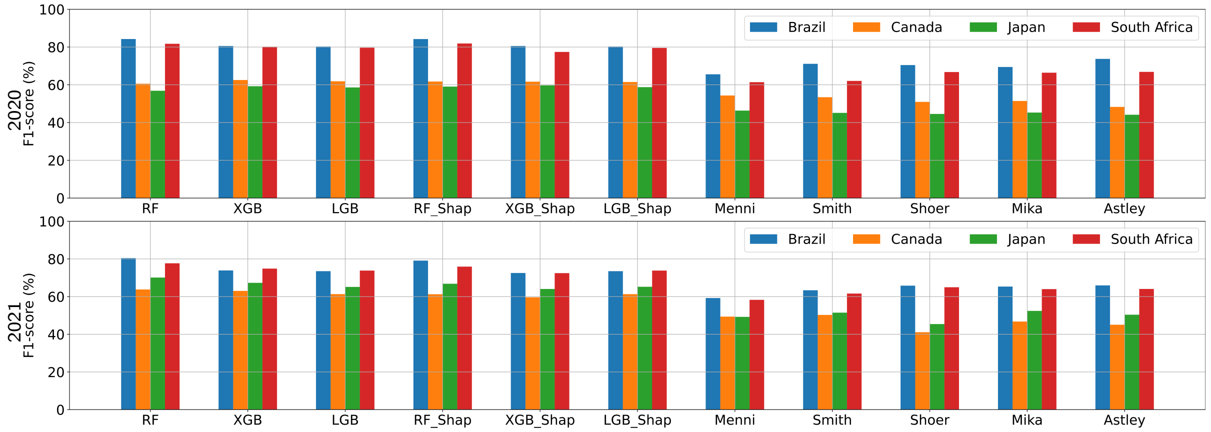

Figure SM1: F_1-scores and the 95% CIs for 2020 and 2021 generated by the various COVID-19 detection methods.

Table SM1

F_1-scores and the 95% CIs yielded by various COVID-19 detection methods for the four countries and for 2020 and 2021.

Year	Method	Brazil	Canada	Japan	South Africa
2020	RF	84.24 (84.19 - 84.29)	60.57 (59.96 - 61.17)	56.84 (55.96 - 57.72)	81.74 (81.54 - 81.94)
	XGB	80.56 (80.50 - 80.62)	62.53 (61.98 - 63.09)	59.24 (58.36 - 60.13)	80.19 (79.96 - 80.42)
	LGB	80.28 (80.22 - 80.33)	61.87 (61.36 - 62.38)	58.60 (57.76 - 59.43)	79.66 (79.45 - 79.87)
	RF_SHAP	84.23 (84.17 - 84.28)	61.72 (61.12 - 62.31)	59.05 (58.27 - 59.83)	81.88 (81.68 - 82.09)
	XGB_SHAP	80.56 (80.51 - 80.62)	61.65 (61.12 - 62.17)	59.70 (58.82 - 60.57)	77.41 (77.19 - 77.64)
	LGB_SHAP	80.26 (80.20 - 80.31)	61.48 (60.88 - 62.07)	58.76 (57.91 - 59.61)	79.54 (79.34 - 79.75)
	Menni [6]	65.56 (65.48 - 65.64)	54.33 (53.66 - 54.99)	46.33 (45.33 - 47.33)	61.39 (61.07 - 61.70)
	Smith [5]	71.11 (71.05 - 71.18)	53.43 (52.85 - 54.01)	45.12 (44.42 - 45.82)	62.06 (61.80 - 62.32)
	Shoer [16]	70.45 (70.39 - 70.52)	50.95 (50.37 - 51.54)	44.57 (43.86 - 45.28)	66.76 (66.52 - 67.00)
	Mika [17]	69.43 (69.37 - 69.49)	51.43 (50.86 - 52.01)	45.29 (44.65 - 45.94)	66.40 (66.13 - 66.68)
	Astley [1]	73.72 (73.65 - 73.78)	48.29 (47.58 - 49.00)	44.13 (43.32 - 44.93)	66.85 (66.61 - 67.09)
2021	RF	80.43 (80.39 - 80.47)	63.80 (63.52 - 64.08)	70.11 (69.84 - 70.37)	77.69 (77.53 - 77.85)
	XGB	73.89 (73.85 - 73.94)	63.03 (62.75 - 63.32)	67.31 (67.03 - 67.60)	74.87 (74.69 - 75.05)
	LGB	73.50 (73.45 - 73.54)	61.32 (61.04 - 61.61)	65.14 (64.87 - 65.41)	73.82 (73.64 - 74.00)
	RF_SHAP	79.11 (79.07 - 79.15)	61.26 (60.97 - 61.56)	66.83 (66.58 - 67.08)	75.92 (75.75 - 76.11)
	XGB_SHAP	72.53 (72.49 - 72.58)	59.63 (59.33 - 59.93)	64.05 (63.77 - 64.34)	72.46 (72.27 - 72.64)
	LGB_SHAP	73.50 (73.45 - 73.54)	61.30 (61.01 - 61.59)	65.22 (64.96 - 65.48)	73.82 (73.64 - 74.00)
	Menni [6]	59.24 (59.18 - 59.31)	49.38 (49.02- 49.74)	49.24 (49.16 - 49.83)	58.28 (58.06 - 58.50)
	Smith [5]	63.37 (63.32 - 63.42)	50.28 (49.99 - 50.57)	51.48 (51.23 -51.74)	61.62 (61.45 - 61.80)
	Shoer [16]	65.81 (65.76 - 65.87)	41.10 (40.84 - 41.36)	45.42 (45.07 - 45.78)	64.97 (64.80 - 65.15)
	Mika [17]	65.33 (65.28 - 65.38)	46.76 (46.40 - 47.12)	52.41 (51.73 - 53.09)	63.98 (63.81 - 64.15)
	Astley [1]	65.95 (65.90 - 66.01)	45.07 (44.74 - 45.40)	50.39 (50.08 - 50.70)	64.06 (63.88 - 64.24)

Table SM2
Performance metrics in % and the 95% CIs obtained by the proposed COVID-19 detection methods for Canada and for 2020 and 2021.

Year	Method	F_1-score	Specificity	Sensitivity	Precision
2020	RF	60.57 (59.97 - 61.18)	**98.80 (98.75 - 98.85)**	48.50 (47.80 - 49.19)	**80.97 (80.29 - 81.66)**
	XGB	**62.54 (61.99 - 63.09)**	97.96 (97.89 - 98.03)	**54.41 (53.71 - 55.10)**	73.77 (73.07 - 74.47)
	LGB	61.87 (61.36 - 62.39)	98.17 (98.11 - 98.24)	52.65 (52.03 - 53.27)	75.24 (74.54 - 75.95)
	RF SHAP	61.72 (61.13 - 62.32)	98.43 (98.37 - 98.50)	51.39 (50.67 - 52.11)	77.57 (76.83 - 78.31)
	XGB SHAP	61.65 (61.13 - 62.17)	97.79 (97.72 - 97.86)	54.03 (53.37 - 54.69)	72.03 (71.27 - 72.79)
	LGB SHAP	61.48 (60.88 - 62.08)	98.05 (97.97 - 98.12)	52.72 (52.01 - 53.42)	74.00 (73.20 - 74.80)
2021	RF	**63.81 (63.53 - 64.08)**	**98.76 (98.73 - 98.79)**	52.03 (51.71 - 52.35)	**82.53 (82.15 - 82.92)**
	XGB	63.04 (62.75 - 63.32)	98.18 (98.15 - 98.22)	**53.48 (53.16 - 53.79)**	76.82 (76.43 - 77.21)
	LGB	61.33 (61.04 - 61.61)	98.18 (98.14 - 98.22)	51.41 (51.08 - 51.73)	76.05 (75.65 - 76.46)
	RF SHAP	61.27 (60.97 - 61.57)	98.57 (98.53 - 98.60)	49.82 (49.48 - 50.15)	79.64 (79.20 - 80.08)
	XGB SHAP	59.63 (59.33 - 59.93)	98.11 (98.07 - 98.14)	49.66 (49.32 - 50.01)	74.68 (74.29 - 75.08)
	LGB SHAP	61.33 (61.03 - 61.62)	98.18 (98.14 - 98.22)	51.40 (51.07 - 51.73)	76.07 (75.66 - 76.48)

Table SM3
Performance metrics in % and the 95% CIs obtained by the proposed COVID-19 detection methods for Japan and for 2020 and 2021.

Year	Method	F_1-score	Specificity	Sensitivity	Precision
2020	RF	56.85 (55.97 - 57.73)	**98.89 (98.82 - 98.97)**	43.33 (42.45 - 44.21)	**83.22 (82.12 - 84.31)**
	XGB	59.25 (58.36 - 60.13)	97.82 (97.72 - 97.92)	49.51 (48.57 - 50.44)	74.17 (73.04 - 75.30)
	LGB	71.54 (71.24 - 71.85)	98.23 (98.19 - 98.26)	63.99 (63.61 - 64.38)	81.16 (80.81 - 81.50)
	RF SHAP	59.06 (58.28 - 59.84)	97.95 (97.85 - 98.04)	48.86 (48.04 - 49.68)	75.03 (73.96 - 76.09)
	XGB SHAP	59.70 (58.83 - 60.58)	97.82 (97.73 - 97.91)	50.05 (49.13 - 50.98)	74.31 (73.23 - 75.39)
	LGB SHAP	**71.55 (71.25 - 71.85)**	98.21 (98.17 - 98.24)	**64.09 (63.72 - 64.46)**	81.03 (80.68 - 81.38)
2021	RF	70.11 (69.85 - 70.38)	**98.95 (98.93 - 98.98)**	59.24 (58.91 - 59.56)	**85.94 (85.61 - 86.27)**
	XGB	67.32 (67.03 - 67.60)	98.48 (98.44 - 98.51)	57.92 (57.58 - 58.25)	80.42 (80.03 - 80.81)
	LGB	80.28 (80.22 - 80.34)	78.53 (78.43 - 78.63)	**79.37 (79.29 - 79.44)**	81.22 (81.14 - 81.30)
	RF SHAP	66.80 (66.54 - 67.06)	98.55 (98.52 - 98.58)	56.91 (56.58 - 57.25)	80.92 (80.57 - 81.28)
	XGB SHAP	64.06 (63.77 - 64.34)	98.40 (98.36 - 98.43)	54.13 (53.80 - 54.47)	78.50 (78.09 - 78.90)
	LGB SHAP	**80.26 (80.21 - 80.32)**	78.53 (78.42 - 78.64)	79.34 (79.26 - 79.41)	81.21 (81.13 - 81.30)

Table SM4
Performance metrics in % and the 95% CIs obtained by the proposed COVID-19 detection methods for South Africa and 2020 and 2021.

Year	Method	F_1-score	Specificity	Sensitivity	Precision
2020	RF	81.75 (81.54 - 81.95)	**92.55 (92.38 - 92.72)**	78.06 (77.75 - 78.38)	85.83 (85.54 - 86.12)
	XGB	80.19 (79.96 - 80.42)	91.30 (91.13 - 91.48)	77.03 (76.69 - 77.38)	83.66 (83.36 - 83.95)
	LGB	79.66 (79.45 - 79.88)	91.23 (91.06 - 91.40)	76.27 (75.95 - 76.58)	83.41 (83.13 - 83.69)
	RF SHAP	**81.89 (81.68 - 82.10)**	92.53 (92.36 - 92.70)	**78.31 (78.01 - 78.62)**	**85.84 (85.55 - 86.13)**
	XGB SHAP	77.42 (77.19 - 77.64)	90.57 (90.36 - 90.77)	73.49 (73.14 - 73.84)	81.83 (81.50 - 82.17)
	LGB SHAP	79.55 (79.35 - 79.75)	91.25 (91.08 - 91.42)	76.07 (75.77 - 76.36)	83.40 (83.12 - 83.68)
2021	RF	**77.70 (77.54 - 77.86)**	**91.83 (91.73 - 91.93)**	**72.47 (72.24 - 72.70)**	**83.76 (83.57 - 83.95)**
	XGB	74.87 (74.69 - 75.05)	90.08 (89.96 - 90.20)	70.06 (69.83 - 70.29)	80.41 (80.18 - 80.64)
	LGB	73.83 (73.65 - 74.01)	90.10 (89.98 - 90.22)	68.50 (68.25 - 68.74)	80.08 (79.86 - 80.31)
	RF SHAP	75.68 (75.52 - 75.84)	90.70 (90.57 - 90.82)	70.63 (70.42 - 70.84)	81.53 (81.29 - 81.76)
	XGB SHAP	72.46 (72.28 - 72.64)	89.52 (89.41 - 89.63)	67.07 (66.82 - 67.32)	78.81 (78.59 - 79.03)
	LGB SHAP	73.82 (73.64 - 74.00)	90.14 (90.02 - 90.26)	68.44 (68.19 - 68.69)	80.13 (79.91 - 80.36)
